# Automated Radiosynthesis of [^18^F]FluoFAPI and Its Dosimetry and Single Acute Dose Toxicological Evaluation

**DOI:** 10.3390/ph17070833

**Published:** 2024-06-25

**Authors:** Jason A. Witek, Allen F. Brooks, Sahil M. Kapila, Wade P. Winton, Jenelle R. Stauff, Peter J. H. Scott, Benjamin L. Viglianti

**Affiliations:** 1 Department of Radiology, University of Michigan Medical School, Ann Arbor, MI 48109, USA; kilsoo@umich.edu (J.A.W.); wwinton@radiopharmacy.com (W.P.W.); jrstauff@med.umich.edu (J.R.S.); pjhscott@med.umich.edu (P.J.H.S.); 2 Department of Chemistry, University of Michigan, Ann Arbor, MI 48109, USA; smkapila@umich.edu; 3 The Interdepartmental Program in Medicinal Chemistry, University of Michigan College of Pharmacy, Ann Arbor, MI 48109, USA

**Keywords:** FAPI, fibroblast activation protein, cancer-associated fibroblast, radioflourination

## Abstract

Background: Cancer-associated fibroblasts have become a new target for therapy. Fibroblasts present within malignancies express the fibroblast activation protein (FAP). Inhibitors to FAP (FAPI) are small molecules recently developed as a theranostic agents for imaging and radiotherapy. All currently used FAPI rely on a linker–chelator complex attached to the ‘inhibitor’. We describe a new automated method of the direct attachment of the radioisotope to the inhibitor, resulting in a >50% MW reduction with the hope of an improved tumor-to-background ratio and tumor uptake. Methods: [^18^F]FluroFAPI was developed from a Sn precursor. This allowed for subsequent automated radioflourination. We obtained the biodistribution of [^18^F]FluroFAPI in rats, performed estimated human radiation dosimetry, and performed a 100× expected single dose toxicology analysis for eventual first-in-human experiments. Results: The synthesis of the Sn precursor for FluorFAPI and the automated synthesis of [^18^F]FluroFAPI was demonstrated. [^18^F]FluroFAPI had favorable estimated human radiation dosimetry, and demonstrated no adverse effects when injected at a dose of 100× that planned for [^18^F]FluroFAPI. Conclusions: With the successful development of an automated synthesis of [^18^F]FluroFAPI, first-in-human testing can be planned with the hope of an improved tumor-to-background performance compared to other FAPI agents.

## 1. Introduction

**Figure 1:**
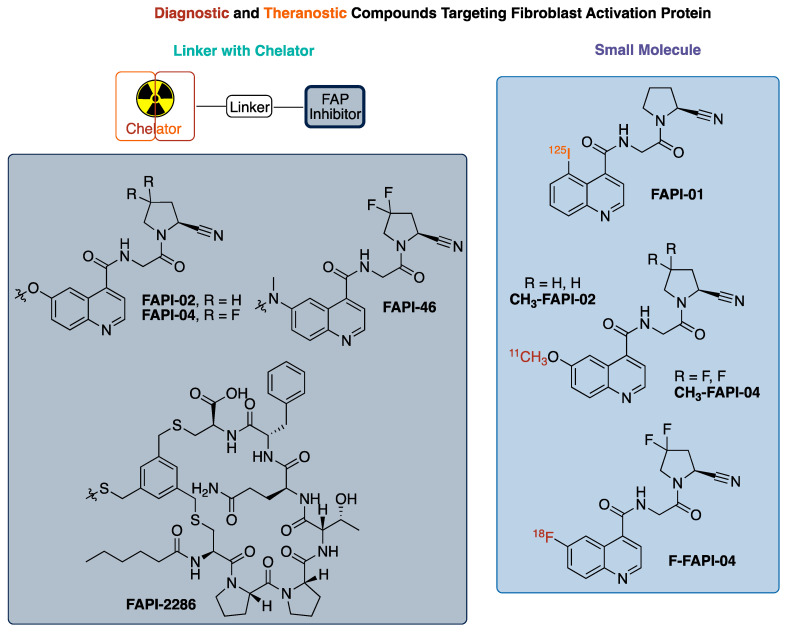
Summary of radiolabeled FAP (fibroblast activation protein) inhibitors.

In 2020, over 600,000 Americans passed away from cancer (CDC) making it the second leading cause of death in the United States. Fibroblasts found in stromal cells are required to support tumor growth with their involvement differing by tumor type, but their presence in the tumor microenvironment is nearly ubiquitous in solid tumor cancers [1]. Several difficult-to-treat cancers have these cancer-associated fibroblasts (CAFs), resulting in these cells being targets for both detection and treatment [1,2]. Specifically, CAFs express a transmembrane protein called fibroblast activation protein (FAP) that is involved in tissue remodeling and tumor growth. In recent years, FAPs have become a target for the development of radiolabeled compounds that could be used as theranostic pairs, where the selection of a radioisotope for labeling provides an imaging agent for detection and staging or for treatment [3]. To date, the majority of developed radiolabeled FAP inhibitor compounds have a general structural layout consisting of a FAP inhibitor pharmacophore [4,5], a linker, and a chelator (Figure 1) [1,2,6]. There are very few small molecule radiolabeled FAP inhibitors that have been reported in the literature that do not use the linker–chelator construct [7,8]. 

FAP, a transmembrane protein, is a serine protease expressed on fibroblasts in up to 90% of epithelial tumors with limited expression in normal tissues, unless undergoing wound healing [4,9,10]. Additionally, some tumors from mesenchymal origin express FAPs, with sarcoma and mesothelioma being the most notable [9,11,12]. Given this expression, FAPs have become a target for radiotherapeutic localization. As a result, multiple families of ligands/inhibitors of FAP (FAPI) have been developed and tested as therapeutics [13,14]. Most of these inhibitors share a similar scaffold, with substitution variations to enhance binding properties or improve pharmacokinetics, as seen in Figure 1 [4]. The testing of these inhibitors as pharmacologic agents (and FAP-directed antibodies) for the treatment of cancer has had limited clinical success to date. However, these inhibitors do accumulate in tumors, leading to interest in their use for diagnostic imaging and as a radiotherapeutic, representing a theranostic pair [4,15,16].

The quinoline-based FAP targeted radiotracer, FAPI-02, -04, -46, has demonstrated encouraging results with a high uptake in >80 patients across a variety of cancers. It was observed that modifications at the 6-position of the quinoline ring had minimal impact on FAP binding [4,5]. Building off the 6-methoxy modification, the investigators utilized a linkage at that position to connect the pharmacophore for FAP to DOTA for the chelation of a radiometal/metal, either ^68^Ga [1,2] or [^18^F]AlF [17,18,19,20], with the choice not affecting tumor uptake in xenograft models. 

The cyclic peptide FAP-2286 represents an alternative to the scaffold of FAPI-02, 04, -46. FAP-2286 was developed to increase the biological half-life, hoping to slow the washout from target tissue, and increase the radiation dose to the tumor. FAP-2286 features a DOTA chelator linked to the cyclic peptide required for targeting FAP. This resulted in a similar uptake to FAPI-46 from injection at 3 h for both the Ga and Lu agents. Although the absolute ^177^Lu uptake at 24 and 72 h was increased in the tumor with FAP-2286, the tumor-to-kidney ratio was better for FAPI-46 at 3 h (21.0 vs. 9.6), identical at 24 h (12.7 vs. 13.1), and better for FAP-2286 at 72 h (27.3 vs. 8.18). Similarly, [^68^Ga]-FAPI-46 had a similar uptake in the tumor to FAP-2286, but FAPI-46 exhibited a faster clearance in the background tissue, providing an improved tumor-to-background ratio of 3–5 times more than that of FAP-2286 at early time points [6]. Consequently, the smaller molecular size of FAPI-02, -04, -46 compared to FAP-2286 offers advantages for the imaging and detection of cancer that express FAPs, and therapeutic advantages are also potentially present if the tumor-to-background ratio is optimized for the decay time of the therapeutic radioisotope, thus avoiding unnecessary radiation doses to healthy tissue and organs.

**Figure 2:**
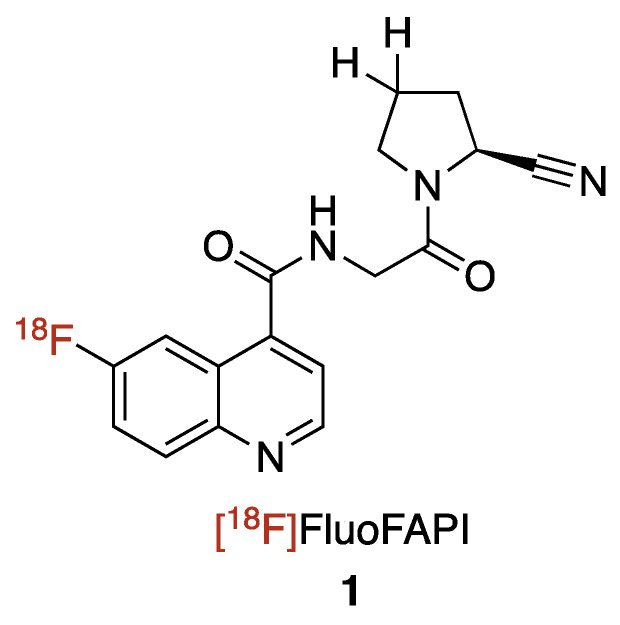
Chemical structure of [^18^F]FluoFAPI.

We are interested in developing and implementing a small molecular FAP inhibitor-focused radiochemistry project with the end goal being the translation of the radioligand to the clinic. We decided to focus on a small molecule ^18^F-radiolabeled compound FAP inhibitor vs. the more commonly reported chelator containing FAP inhibitors, for several reasons, as follows: (1) ^18^F possesses physical properties that lends to it having ideal PET imaging properties (half-life: ~2X ^68^Ga, ~6X ^11^C; imaging resolution (full width at half maximum of 0.54 mm): ~5X ^68^Ga, ~2X ^11^C)^9^; (2) In the US, there is already a nationwide infrastructure in place for manufacturing commercial ^18^F agents; (3) A ^18^F-labeled FAPI agent would be expected to have a better tumor/background targeting with its much lower molecular weight (~1/2 that of the reported chelator-containing agents); (4) the logP of the agent will be improved and in line with typical small molecule PET imaging agents through not having the polar groups (amines and carboxylic acids) found in chelators; and (5) ^18^F radiochemistry methods would offer the potential for a common precursor to be used for not only ^18^F labeling, but also labeling with other radioisotopes such as ^131^I (for beta therapy) or ^211^At (for alpha therapy). Herein, we report the development of [^18^F]FluoFAPI, as well as its automated radiosynthesis, radiation dosimetry, and single acute dose toxicological evaluation (Figure 2).

## 2. Results and Discussion

### 2.1. Organic Syntheses of [^19^F]FluoFAPI 1 and Me_3_SnFAPI 2

**Scheme 1:**
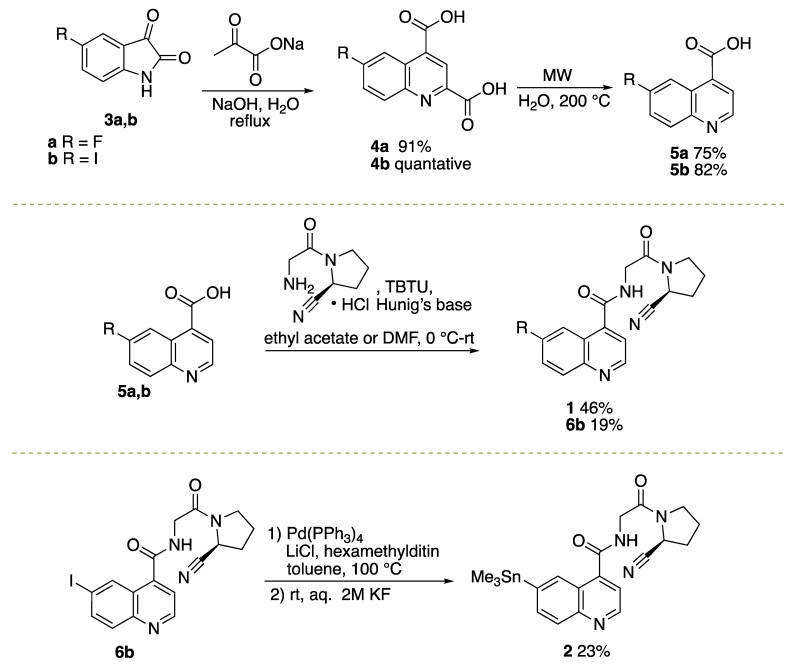
Synthetic routes to Me_3_SnFAPI **2** and FluoFAPI Standard **1**.

Commercially available isatins **3a, b** were converted into quinoline-2,4-dicarboxylic acids **4a, b** under Pfitzinger reaction conditions [4,5], followed by microwave-mediated decarboxylation to afford quinoline-4-carboxylic acids **5a, b** (Scheme 1) [21]. FluoFAPI **1** and iodo quinoline **6b** were generated through peptide coupling reactions in either ethyl acetate or DMF [4,5]. Iodo quinoline **6b** was converted to Me_3_SnFAPI **2** using conditions that are consistent with previous reports for stannylation or heterocycles [22]. In preparation for the radiosynthesis of [^18^F]FluoFAPI, various analytical and semi-preparative HPLC conditions were explored. It was found that FluoFAPI **1** and Me_3_SnFAPI **2** were separable using either an analytical HPLC column (Luna 5µ C18(2), 150 × 4.6; Buffer: 20% MeCN, 10 mM NH_4_OAc, pH 5.0; Flow rate: 2 mL/min @ 40 °C; UV = 254 nm) or a semi-preparative HPLC column (Gemini 5µ NXC18 110 Å, 250 × 10; Buffer: 55% MeCN, 10 mM NH_4_HCO_3_, pH 10.0; Flow rate: 4 mL/min @ rt; UV = 254 nm).

### 2.2. Radiosynthesis of [^18^F]FluoFAPI

**Scheme 2:**
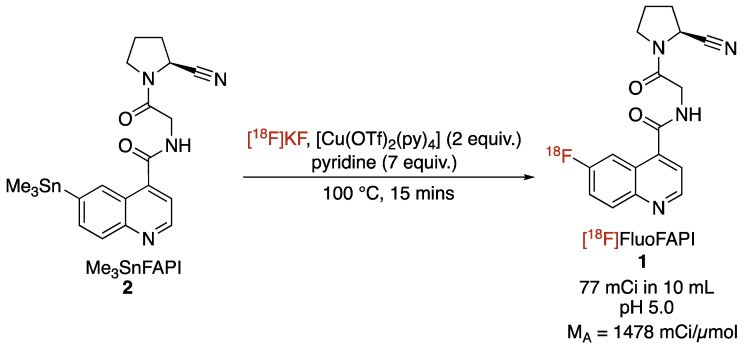
Radiosynthesis of [^18^F]FluoFAPI.

The automated radiosynthesis of [^18^F]FluoFAPI **1** was carried out in a TRACERLab FX_FN_ synthesis module (Scheme 2). Previously reported conditions for the copper-mediated [^18^F]fluorination of aryl stannanes were used [22]. After the radiolabeling was complete, the reaction mixture was purified in the synthesis module utilizing semi-preparative HPLC, the mixture was then trapped/released on a Waters C18 1cc extraction disk, and was then formulated for injection (10% ethanol/0.9% saline solution). [^18^F]FluoFAPI was synthesized to >99% radiochemical purity, with a 4.3% activity yield (77 mCi; 73 min from EOB; molar activity of 1478 mCi/µmol).

### 2.3. Biodistribution and Dosimetry

Real-time measurements of [^18^F]FluoFAPI uptake in 16 Sprague Dawley rats (male = 8, female = 8) were performed over 4 time points (10 min, 20 min, 60 min, and 120 min) post-injection. In addition, Sprague Dawley rats (2 male, 2 female) were dynamically imaged in a small animal PET scanner with bladder in frame to provide dose measure in urine. It was found that at all time points, uptakes in both the liver and small intestine were the highest (liver = 0.4987 ± 0.0620%ID/g at 10 min p.i., 0.3710 ± 0.0308%ID/g at 20 min p.i., 0.1958 ± 0.019 7%ID/g at 60 min p.i., and 0.1088 ± 0.0079%ID/g at 120 min p.i.; small intestine = 0.6419 ± 0.0777%ID/g at 10 min p.i., 0.5997 ± 0.0634%ID/g at 20 min p.i., 0.4855 ± 0.0581%ID/g at 60 min p.i., and 0.4632 ± 0.0516%ID/g at 120 min p.i.). Additionally, it was found that uptake in the kidney of [^18^F]FluoFAPI was 0.8577 ± 0.1446%ID/g at 10 min p.i., 1.118 ± 0.1473%ID/g at 20 min p.i., 0.6052 ± 0.1086%ID/g at 60 min p.i., and 0.4346 ± 0.1225%ID/g at 120 min p.i.

The amount of radiation dose to different target organs in mSv/MBq can be seen in the table below (Table 1). The human radiation dose estimates were calculated from the biodistribution studies in male and female Sprague Dawley rats over four time points. Dosimetry calculations were accomplished using OLINDA/EXM 2.2.0 in a manner consistent with previous reports, to provide an expected human dose from a [^18^F]FluoFAPI PET scan of 1.85 × 10^−2^ mSv/MBq [23,24]. 

### 2.4. Single Acute Dose Toxicological Evaluation

A single dose toxicity study was carried out using 10 male and 10 female Sprague Dawley rats using a human equivalent dose greater in injected mass then the expected human dose for a PET imaging scan (33.5 µg/kg versus 0.335 µg/kg). No adverse effects were found in the heart, liver, kidney, spleen, and brain. In addition, there were no adverse effects found in any hematology parameters (ex. red cell count, etc.) or biochemistry parameters (ex. albumin, etc.); see Supplementary Materials for more details.

## 3. Materials and Methods

### 3.1. Organic Chemistry

#### 3.1.1. General 

All the chemicals were purchased from commercially available suppliers and were used without purification. Automated flash chromatography was performed with the Biotage Isolera Prime system. High-performance liquid chromatography (HPLC) was performed using a Shimadzu LC-2010A HT system equipped with a Bioscan B-FC-1000 radiation detector. ^1^H and ^13^C NMR spectra were obtained using Varian 500 apparatus (400 MHz for ^1^H NMR and 100 MHz for ^13^C NMR) in DMSO-d_6_ or CDCl_3_, unless otherwise indicated; δ in ppm relative to tetramethylsilane (δ = 0), *J* in Hz. Mass spectrometry (HRMS) was performed using an Agilent 6520 Accurate-Mass Q-TOF LC/MS spectrometer using ESI ionization with less than a 5 ppm error for all HRMS analyses.

#### 3.1.2. Syntheses

##### General Procedure for the Synthesis of Quinoline-2,4-dicarboxylic Acid Derivatives

To a round bottom flask was added isatin derivative **3a,b** (1 mmol) and H_2_O (10 mL) at room temperature. NaOH (240 mg, 6 mmol) was added portion-wise, followed by the addition of sodium pyruvate (176 mg, 1.6 mmol). The reaction mixture was heated to reflux and allowed to stir for 48 h. Upon cooling to room temperature, the pH of the reaction mixture was adjusted to a pH of 2 using 1 M HCl. The resulting precipitate was filtered; washed with water, dichloromethane, and hexanes; and dried in vacuo to afford the desired compounds **4a,b**. 

##### 6-Fluoroquinoline-2,4-dicarboxylic Acid (**4a**). The desired product was obtained as a light-tan solid (2.58 g, 91% yield)

^1^H NMR (400 MHz, DMSO-*d6*) δ 8.60–8.48 (m, 2H), 8.35–8.25 (m, 1H), 7.92–7.78 (m, 1H). 

##### 6-Iodoquinoline-2,4-dicarboxylic Acid (**4b**)

The product was obtained as a light-orange solid (2.5 g, 100% yield). ^1^H NMR (400 MHz, DMSO-*d6*) δ 9.26 (s, 1H), 8.48 (s, 1H), 8.20–8.14 (m, 1H), 8.00–7.95 (m, 1H).

##### General Procedure for the Synthesis of Quinoline-4-carboxylic Acid Derivatives

To a 10 mL microwave vial was added **4a,b** (1 mmol) and H_2_O (4 mL). The reaction was heated to and held at 200 °C in the microwave for 5–10 min. After cooling to room temperature, the reaction mixture was filtered and dried under vacuum to give the desired compound **5a,b**.

##### 6-Fluoroquinoline-4-carboxylic Acid (**5a**)

The compound was obtained as a dark-brown solid (122 mg, 75% yield). ^1^H NMR (400 MHz, DMSO-*d6*) δ 9.03 (d, *J* = 4.0 Hz, 1H), 8.50 (dd, *J* = 11.2, 3.0 Hz, 1H), 8.19 (dd, *J* = 9.3, 5.8 Hz, 1H), 8.02 (d, *J* = 4.4 Hz, 1H), 7.77 (td, *J* = 8.7, 2.9 Hz, 1H); ^19^F NMR (376 MHz, DMSO-*d6*) δ -110.6-(-)110.8 (m, 1F).

##### 6-Iodoquinoline-4-carboxylic Acid (**5b**)

The desired product was afforded as a brown solid (145 mg, 83% yield). ^1^H NMR (400 MHz, DMSO-*d6*) δ 9.18 (d, *J* = 4.0 Hz, 1H), 8.09 (dd, *J* = 8.8, 2.0 Hz, 1H), 7.97 (d, *J* = 4.4 Hz, 1H), 7.88 (d, *J* = 8.8 Hz, 1H).

##### General Procedure for the Synthesis of (S)-N-(2-(2-Cyanopyrrolidin-1-yl)-2-oxoethyl)quinoline-4-carboxamide Derivatives

To a round bottom flask was added compound **5a,b** (1.05 mmol) and DMF (10 mL) at 0 °C along with Hunig’s base (523 μL, 3 mmol). TBTU (1.05 mmol) was added slowly portion-wise and the reaction mixture was allowed to stir for 30 min. TBTU (1.05 mmol) was added portion-wise and the solution was warmed to room temperature. After stirring for 48 h, the reaction mixture was diluted with dichloromethane (10 mL); washed with H_2_O, brine, and sat. aq. LiCl; and was dried over Na_2_SO_4_. The combined organic layers were dried in vacuo and the resulting residue was purified using column chromatography (1–10% MeOH/DCM) to give the desired compounds **1** or **6b**.

##### (S)-N-(2-(2-cyanopyrrolidin-1-yl)-2-oxoethyl)-6-fluoroquinoline-4-carboxamide (FluoFAPI 1)

Compound **1** was obtained as a brown foam (11.9 mg, 46% yield). ^1^H NMR (400 MHz, DMSO-*d6*) δ 9.12 (t, *J* = 6.0 Hz, 1H), 8.98 (d, *J* = 4.0 Hz, 1H), 8.20–8.13 (m, 2H), 7.76 (dt, *J* = 8.0, 4.0 Hz, 1H), 7.63 (d, *J* = 4.0 Hz, 1H), 4.86 (dd, *J* = 7.26, 3.66 Hz, 1H), 4.28–4.12 (m, 2H), 3.77–3.69 (m, 1H), 3.58–3.49 (m, 1H), 2.22–2.00 (m, 4H); ^13^C NMR (100 MHz, DMSO-*d6*) δ 167.9, 167.3, 159.6, 150.2, 145.7, 142.0, 132.7, 120.6, 120.4, 120.3, 119.8, 109.8, 46.8, 45.8, 42.0, 29.9, 25.3; ^19^F NMR (376 MHz, DMSO-*d6*) δ -111.6-(-)111.8 (m, 1F).

##### (S)-N-(2-(2-cyanopyrrolidin-1-yl)-2-oxoethyl)-6-iodoquinoline-4-carboxamide (**6b**)

The desired compound **6b** was afforded as a brown foam (24.5 mg, 71% yield). ^1^H NMR (400 MHz, DMSO-*d6*) δ 9.12 (t, *J* = 5.8 Hz, 1H), 9.00 (d, *J* = 4.4 Hz, 1H), 8.74 (d, *J* = 2.0 Hz, 1H), 8.07 (dd, *J* = 8.8, 2.0 Hz, 1H), 7.85 (d, *J* = 8.8 Hz, 1H), 7.59 (d, *J* = 4.4 Hz, 1H), 4.84 (dd, *J* = 7.4, 3.4 Hz, 1H), 4.20 (qd, *J* = 16.9, 5.89 Hz, 2H), 3.76–3.69 (m, 1H), 3.58–3.47 (m, 1H), 3.42–3.34 (m, 1H), 2.22–2.05 (m, 3H); ^13^C NMR (100 MHz, DMSO-*d6*) δ 167.7, 167.1, 151.3, 147.2, 141.1, 138.7, 134.7, 131.7, 126.3, 120.3, 119.8, 94.7, 46.8, 45.8, 42.0, 29.9, 25.3. 

##### Synthesis of (S)-N-(2-(2-cyanopyrrolidin-1-yl)-2-oxoethyl)-6-(trimethylstannyl)quinoline-4-carboxamide (Me_3_SnFAPI 2)

To a flame-dried round bottom flask was added Pd(PPh_3_)_4_ (77.4 mg, 0.067 mmol) and LiCl (67.8 mg, 1.60 mmol). The round bottom flask was then placed under vacuum and backfilled with argon 3X. A solution of **6b** in toluene (3.3 mL) was then added, followed by the drop-wise addition of hexamethylditin (567 mg, 1.73 mmol). The reaction mixture was then heated to 100 °C and stirred for 16 h. Upon cooling to room temperature, 2 M aqueous KF (1.7 mL) was added and the mixture was allowed to stir for 30 min. The contents of the round bottom flask were then poured over celite, washed with brine, dried over Na_2_SO_4_, and concentrated in vacuo. After purification using flash column chromatography (1–10% methanol/dichloromethane), the desired product **2** was obtained as a white foam (36.5 mg, 23% yield). ^1^H NMR (400 MHz, DMSO-*d6*) δ 9.04 (t, *J* = 5.4 Hz, 1H), 8.93 (d, *J* = 3.6 Hz, 1H), 8.60–8.46 (m, 1H), 8.04–7.83 (m, 2H), 7.54–7.49 (m, 1H), 4.79 (dd, *J* = 8.0, 4.0 Hz, 1H), 4.27–4.12 (m, 2H), 3.77–3.68 (m, 1H), 3.54 (q, *J* = 8.0 Hz, 1H), 2.25–1.96 (m, 4H), 0.43–0.24 (m, 9H); ^13^C NMR (100 MHz, DMSO-*d6*) δ 167.78, 167.75, 150.6, 148.4, 142.7, 142.2, 136.7, 134.2, 128.6, 124.3, 119.7, 119.4, 46.8, 45.7, 42.0, 30.0, 25.3, −8.67; HRMS (ESI^+^-TOF) (C20H25N4O2Sn) calcd 472.0921 (M + H), found 473.0994.

### 3.2. Radiochemistry

#### 3.2.1. General

Unless otherwise stated, reagents and solvents were commercially available and used without further purification. Sodium chloride, 0.9% USP, and sterile water for injection, USP, were purchased from Hospira; ethanol was purchased from American Regent; HPLC-grade acetonitrile was purchased from Fisher Scientific. Other synthesis components were obtained as follows: sterile filters were obtained from Millipore; sterile product vials were purchased from Hollister-Stier; C18 Sep-Paks were purchased from Waters Corporation. C18 Sep-Paks were flushed with 10 mL of ethanol followed by 10 mL of water prior to use.

#### 3.2.2. Automated Radiosynthesis of [^18^F]FluoFAPI 1

**Figure 3:**
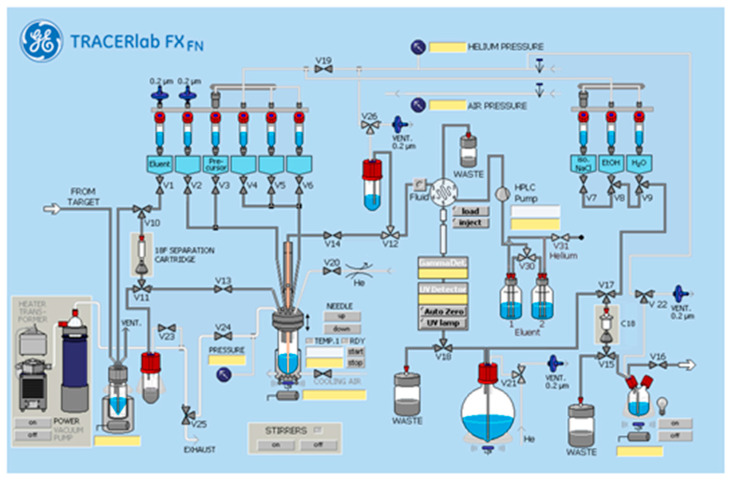
TRACERlab FX_FN_ standard configuration.

[^18^F]FluoFAPI **1** radiosynthesis was automated in a TRACERLab FX_FN_ module (Figure 3). Briefly, cyclotron-produced ^18^F was trapped on a QMA Sep-Pak and eluted into the reactor using KOTf (10 mg) and minimal K_2_CO_3_ (50 µg) in H_2_O (0.5 mL). Azeotropic drying was then carried out using acetonitrile (1 mL) at 100 °C, first under vacuum for 5 min and then under vacuum with argon over pressure for an additional 5 min. To the dried [^18^F]KF was added a solution of the stannane precursor (5.0 mg, 0.01 mmol) in DMA (830 µL) followed by the addition of a solution of [Cu(OTf)_2_(py)_4_] (0.2 M stock solution in DMA, 100 µL, 0.02 mmol) and pyridine (1 M stock solution in DMA, 70 µL, 0.14 mmol). The reaction mixture was then heated to 100 °C and allowed to stir for 15 min. Upon cooling to 50 °C, 2 mL of buffer (20% acetonitrile, 10 mM NH_4_HCO_3_, pH 10) was added and after stirring for an additional 1 min, was transferred to an HPLC loop for injection and purification using semi-preparative chromatography (Gemini 5 µm NXC18 110 Å, 250 × 10 mm, 4 mL/min). The product peak (retention time ~ 20 min) was collected and diluted into 50 mL of MQ H_2_O followed by trapping on a C18 extraction disk. The trapped product was washed with 10 mL of sterile water, eluted with 500 µL of EtOH, and then rinsed with 4.0 mL of saline into the collection vial containing 5.5 mL of saline. The resulting 10 mL solution was then passed through a sterile filter into a sterile 10 mL dose vial. The identity and purity of [^18^F]FluoFAPI **1** was then confirmed using HPLC (Luna C18(2), 150 × 4.6 mm, 5µ, Buffer: 20% acetonitrile, 10 mM NH_4_OAc, pH 5.0, 2 mL/min at 40 °C).

### 3.3. Biodistribution and Dosimetry

The biodistribution of [^18^F]FluoFAPI was evaluated in healthy Sprague Dawley rats at several time points post-injection (10 min, 20 min, 60 min, and 120 min). Rats were put under general anesthesia and were administered doses of [^18^F]FluoFAP through the tail vein. The organs/tissues of interest were harvested after the animals were euthanized at the specified time points. The organs/tissues were then weighed and the gamma ray radiation was counted followed by the calculation of radioactive uptakes, which are reported as percentage of injected dose per gram (%ID/g). 

### 3.4. Single Acute Dose Toxicological Evaluation

Single acute dose toxicological evaluation was carried out at the in vivo facility at Michigan State University. The evaluation was carried out on ten male and ten female Sprague Dawley rats and was compared to control animals. The animals were housed individually in plastic solid-bottom cages with aspen bedding throughout the duration of the study. In addition, the animals had access to fresh water and were provided standard rodent chow in sufficient amounts to ensure ad libitum consumption. After approximately 1 week of acclimation, body weights were recorded. Light anesthesia was carried out using isoflurane, and then blood (~1 mL total) was collected into K_3_EDTA and serum tubes and was then processed for clinical chemistry and complete blood count (CBC) baseline measurements. The test formulation (33.54 µg/kg body weight, 3.12 mL/kg of FluoFAPI) was then delivered as a single intravenous bolus. Clinical observations were recorded daily. Food consumption was calculated on prior dosing (baseline) and on days 2, 7, and 15. Five male and five female rats were euthanized on day 2, and the remaining animals on day 15. Terminal body weights were obtained and terminal blood samples were collected and processed for clinical chemistry and CBC parameters. The heart, liver, kidney, spleen, and brain were removed, and were weighed and fixed in 10% neutral-buffered formalin. Slides were prepared, stained with standard Hematoxylin and Eosin, and reviewed by a qualified veterinary pathologist.

## 4. Conclusions

An automated radiosynthesis of [^18^F]FluoFAPI formulated for clinical use has been reported. The biodistribution and dosimetry in Sprague Dawley rats and the human dose estimates to all major organs were found to be within acceptable levels. Additionally, no adverse effects at a dose greater than the expected dose for a PET study were found.

## Figures and Tables

**Table 1:** Human radiation dose estimates calculated from data collected using Sprague Dawley rats (male and female) at four time points (10, 20, 60, and 120 min); see ESI for biodistribution data used in calculations.

^18^F-FluoFAPI	Gender-Averaged Dose
Target Organ	mSv/MBq
Adrenals	7.11 × 10^−3^
Brain	2.03 × 10^−3^
Breasts	8.25 × 10^−3^
Esophagus	8.16 × 10^−3^
Eyes	4.07 × 10^−3^
Gallbladder Wall	1.09 × 10^−2^
Left colon	1.42 × 10^−2^
Small Intestine	1.96 × 10^−2^
Stomach Wall	9.89 × 10^−3^
Right colon	1.90 × 10^−2^
Rectum	2.62 × 10^−2^
Heart Wall	6.97 × 10^−3^
Kidneys	1.18 × 10^−2^
Liver	6.64 × 10^−3^
Lungs	5.82 × 10^−3^
Ovaries	1.86 × 10^−2^
Pancreas	8.19 × 10^−3^
Prostate	2.32 × 10^−2^
Salivary Glands	8.38 × 10^−3^
Red Marrow	9.49 × 10^−3^
Osteogenic Cells	8.66 × 10^−3^
Spleen	6.62 × 10^−3^
Testes	8.35 × 10^−3^
Thymus	8.98 × 10^−3^
Thyroid	8.62 × 10^−3^
Urinary Bladder Wall	2.27 × 10^−1^
Uterus	3.12 × 10^−2^
Total Body	1.10 × 10^−2^
Effective Dose:	1.85 × 10^−2^ (mSv/MBq)

## Data Availability

Data contained within the article and Supplementary Materials will be made available on request.

## Supplementary Materials

The following supporting information can be downloaded at: https://www.mdpi.com/article/10.3390/ph17070833/s1. Small Molecule Characterization Data, HPLC Traces, Biodistribution Data, and Single Acute Dose Toxicology Data.
