# Supplementary material for: Automated Radiosynthesis of [18F]FluoFAPI and Its Dosimetry and Single Acute Dose Toxicological Evaluation
Source: Pharmaceuticals (Basel). 2024 Jun 25;17(7):833. doi: 10.3390/ph17070833 (PMC11280013; doi:10.3390/ph17070833)
Supplement: Supplementary file 1 [file pharmaceuticals-17-00833-s001.zip › pharmaceuticals-3052418-supplementary.pdf]

Supplementary Materials

To

Automated Radiosynthesis of [^{18}F]FluoFAPI and its' Dosimetry and Single Acute Dose Toxicological Evaluation

Jason A. Witek[†], Sahil M. Kapila[‡], Wade P. Winton[†], Jenelle R. Stauff[†], Peter J. H. Scott[†§], Allen F. Brooks[†*], Benjamin L. Viglianti[†*]

[†]Department of Radiology, University of Michigan Medical School, Ann Arbor, MI 48109, United States

[‡]Department of Chemistry, University of Michigan, Ann Arbor, MI 48109, United States

[§]The Interdepartmental Program in Medicinal Chemistry, University of Michigan College of Pharmacy, Ann Arbor, MI 48109, United States

Content:

1) NMR and other characterization of compounds **4(a,b)**, **5(a,b)**, **1**, **6b** and **2**
2) Radio- and UV-HPLC chromatograms of
   i) Semi-preparative separation of [^{18}F]FluoFAPI **1**
   ii) Final formulated dose [^{18}F]FluoFAPI **1**
   iii) Final formulated dose co-inject [^{18}F]FluoFAPI **1**

3) Biodistribution data of [^{18}F]FluoFAPI **1** in Sprague-Dawley rats
4) Single Acute Dose Toxicological Data

1) NMR and other characterization of compounds **4(a,b)**, **5(a,b)**, **1**, **6b** and **2**.

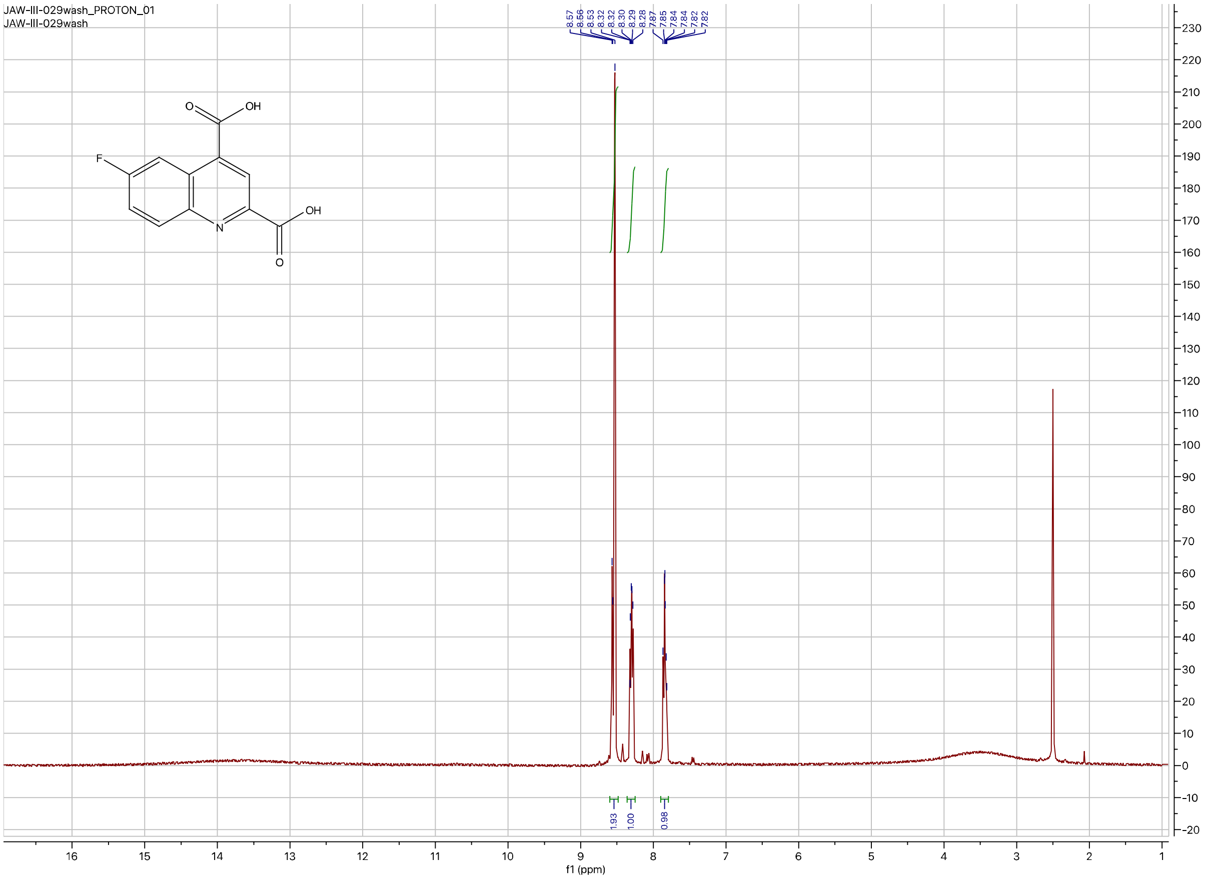

Figure S1. ^{1}H NMR of compound **4a**.

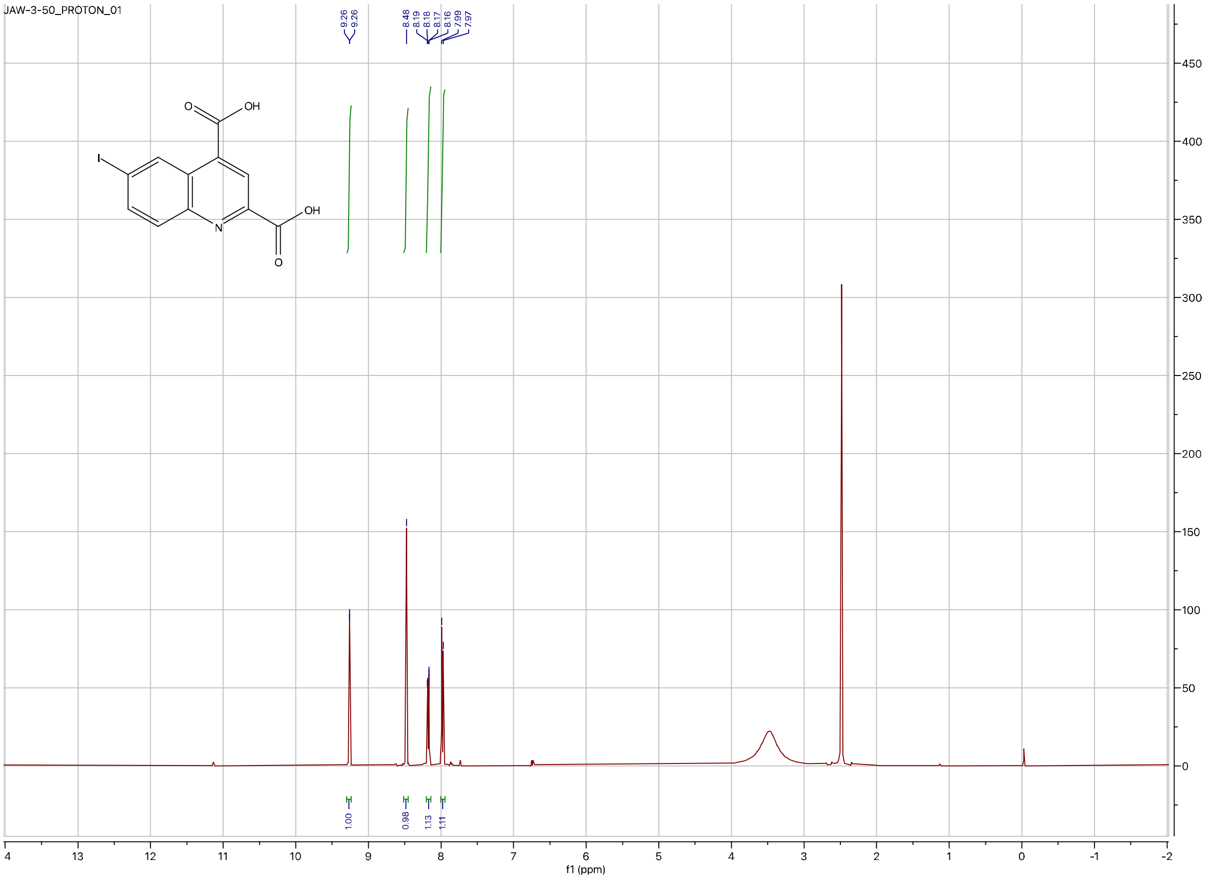

Figure S2. ^{1}H NMR of compound **4b**.

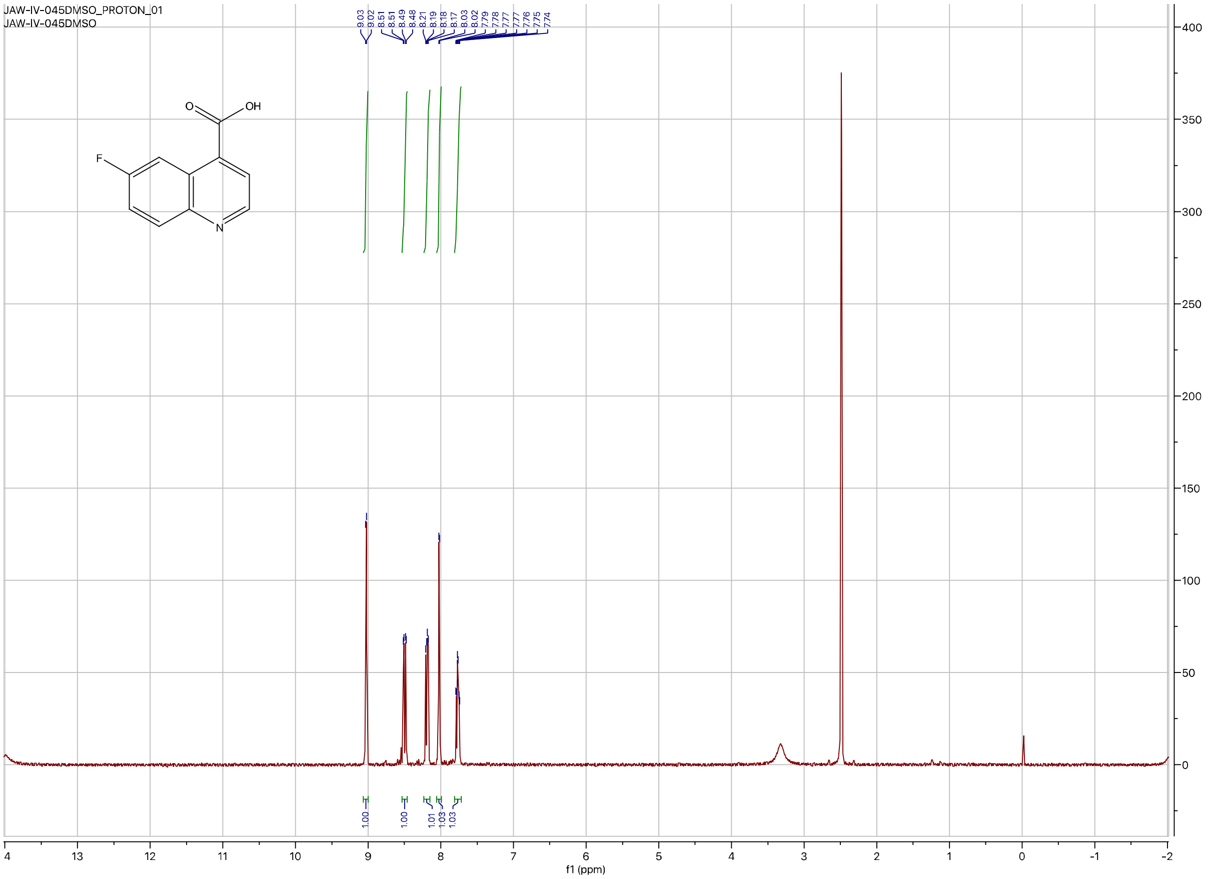

Figure S3. ^{1}H NMR of compound **5a**.

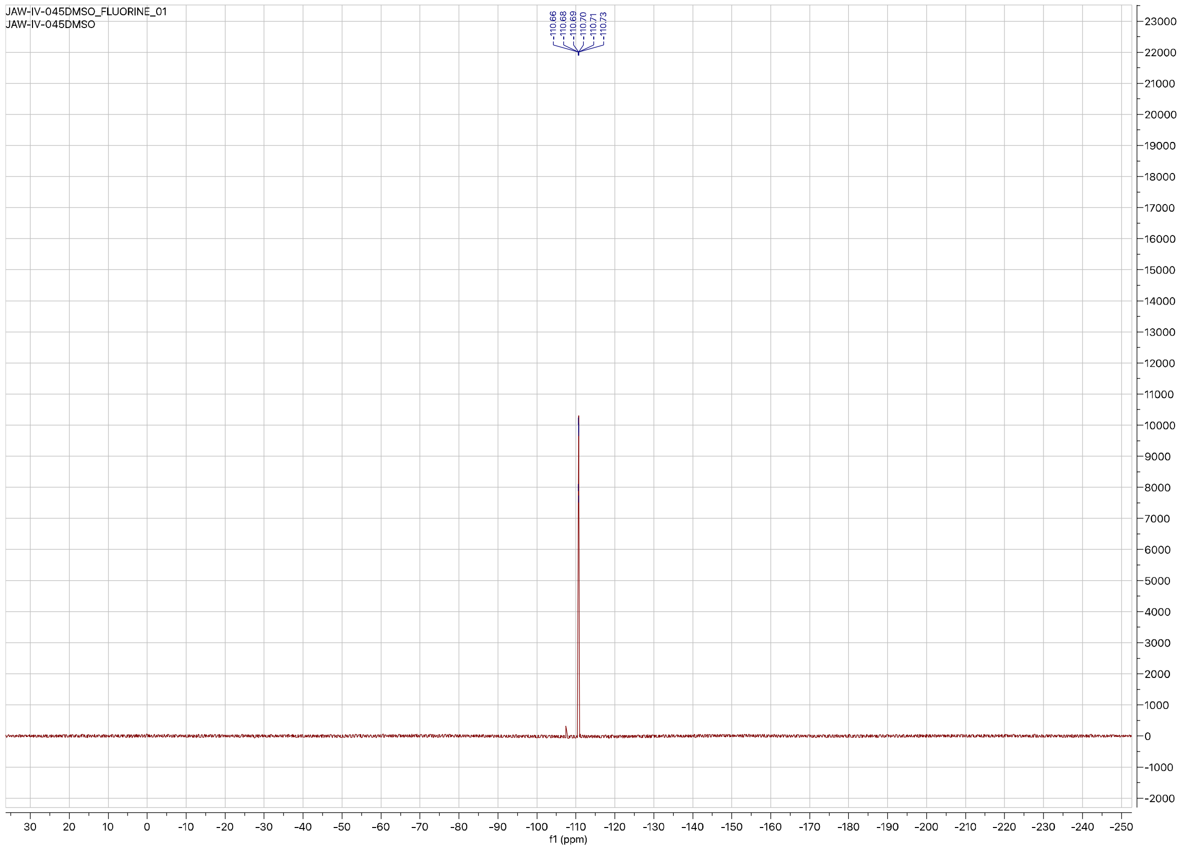

Figure S4. ^{19}F NMR of compound **5a**.

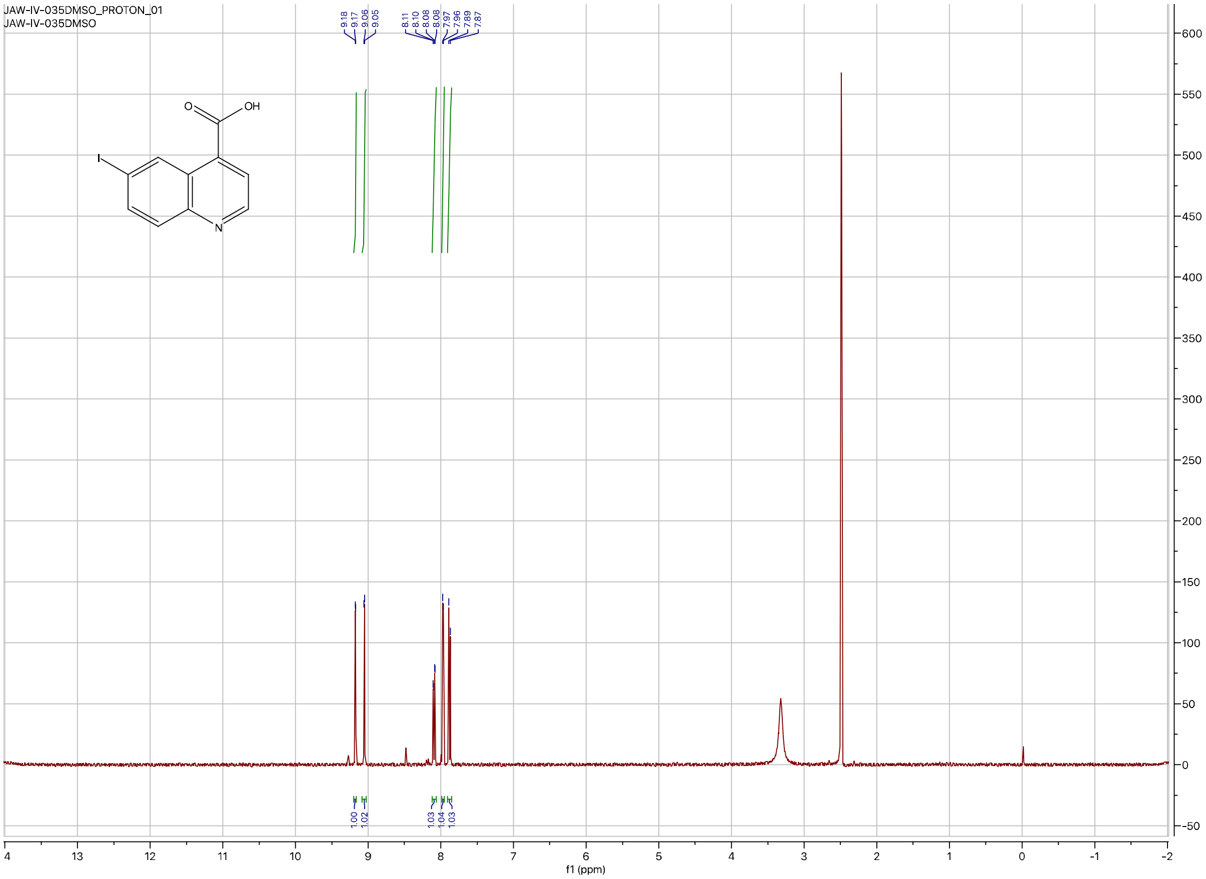

Figure S5. ^{1}H NMR of compound **5b**.

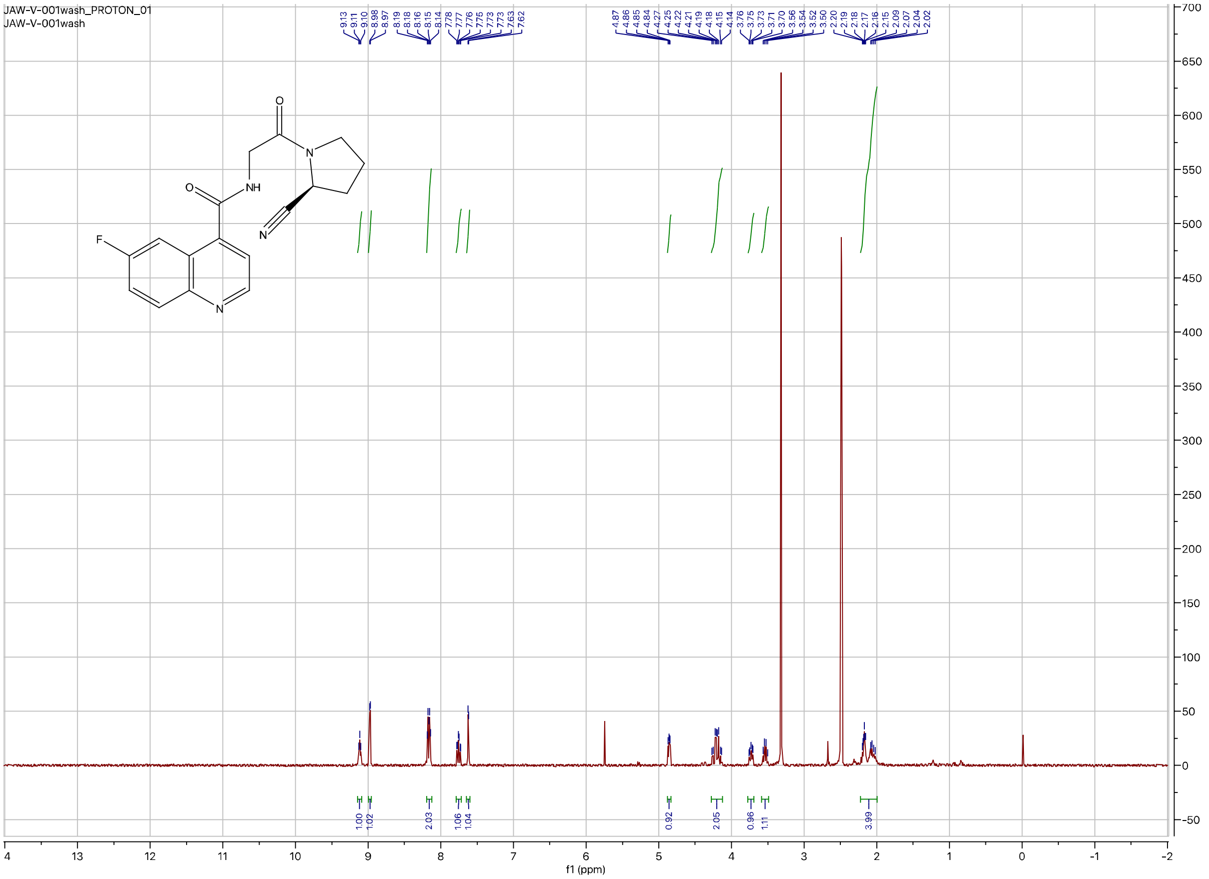

Figure S6. ^{1}H NMR of FluoFAPI **1**.

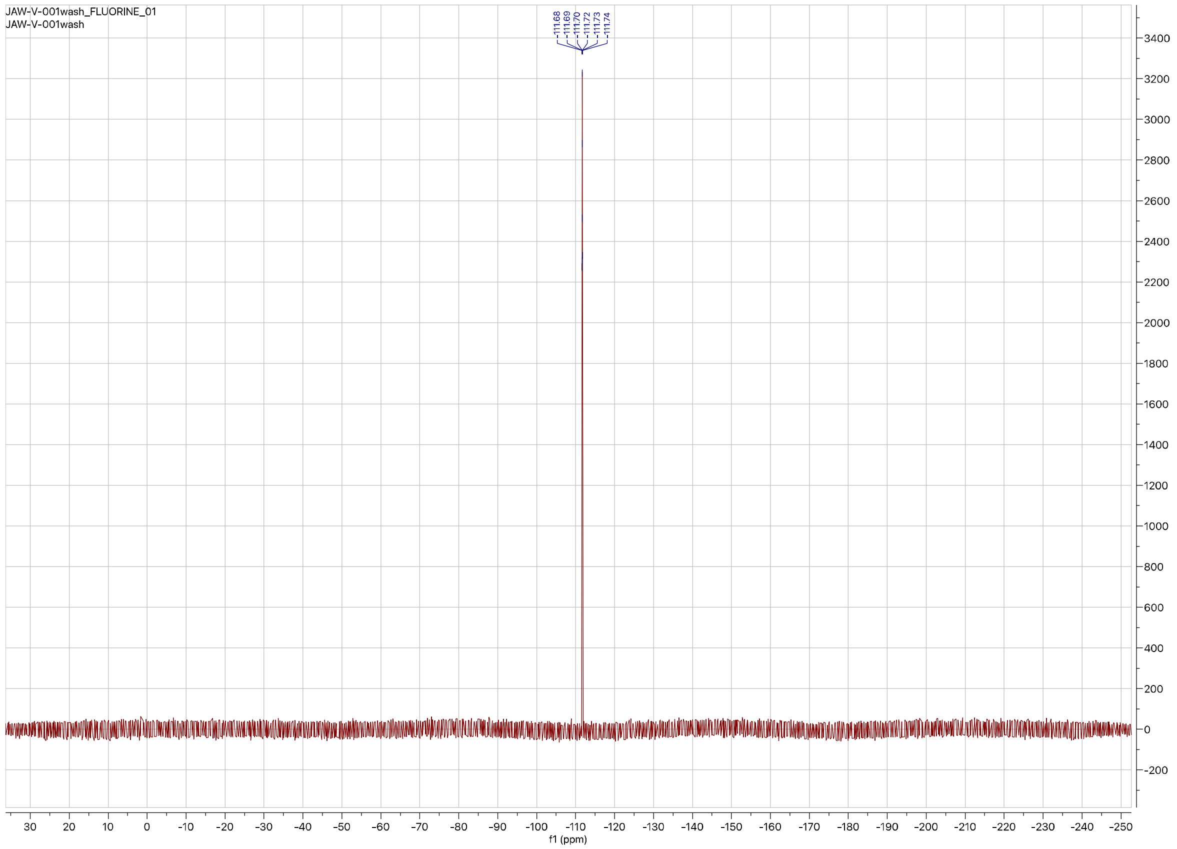

Figure S7. ^{19}F NMR of FluoFAPI **1**.

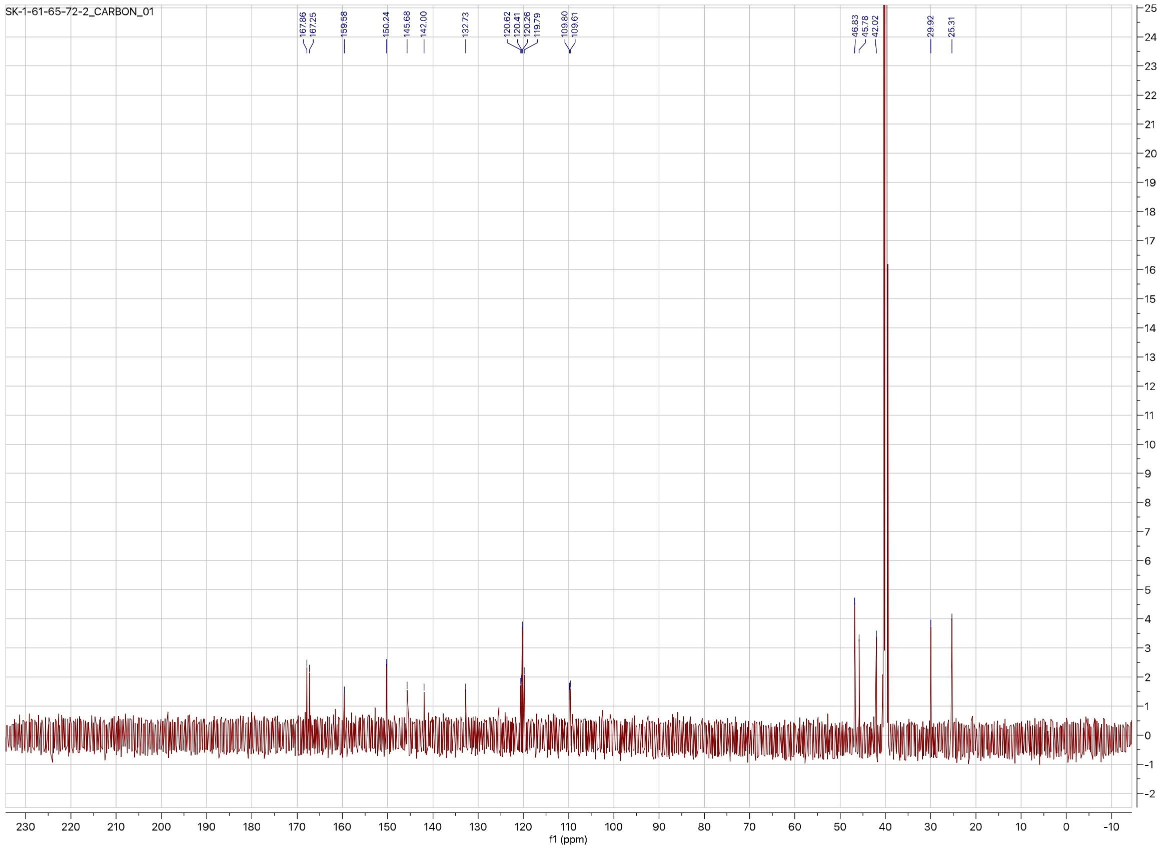

Figure S8. ^{13}C NMR of FluoFAPI **1**.

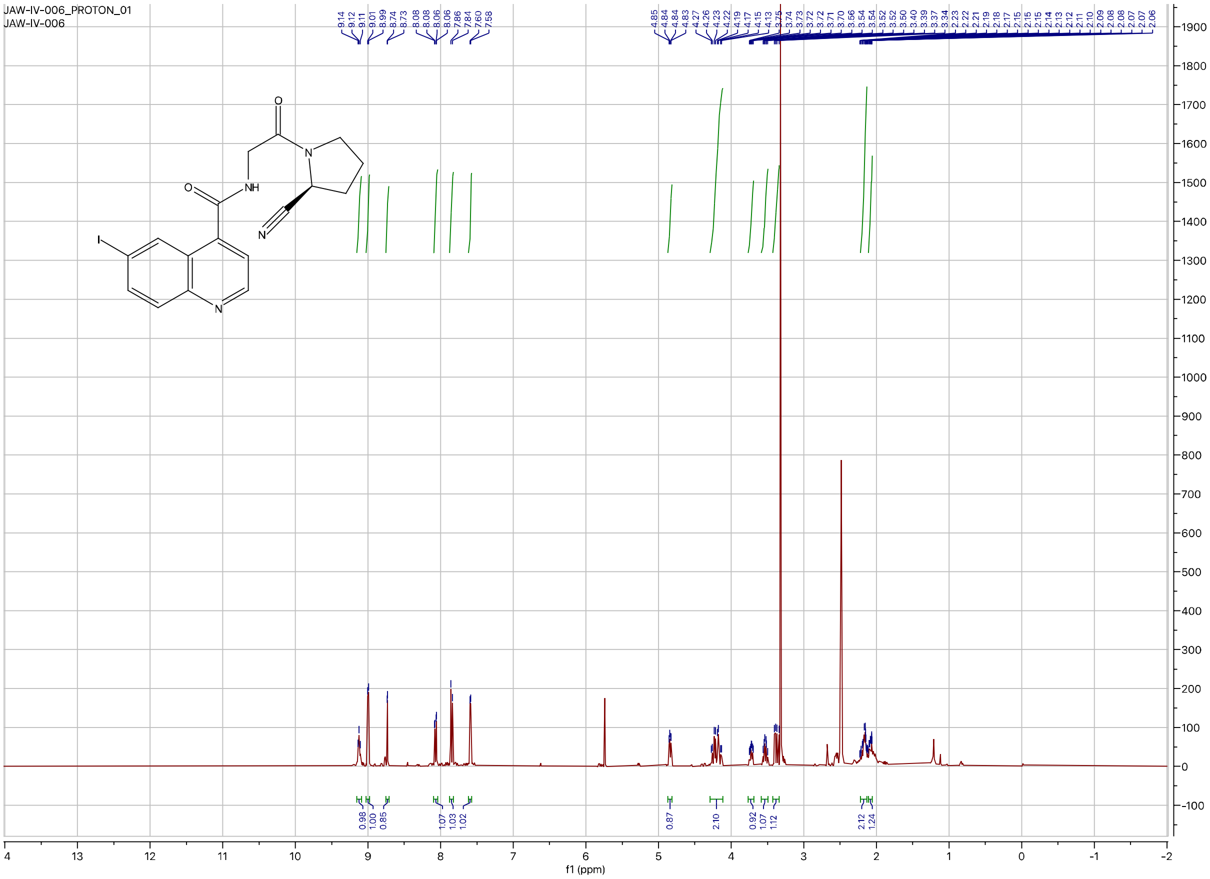

Figure S9. ^{1}H NMR of Compound **6b**.

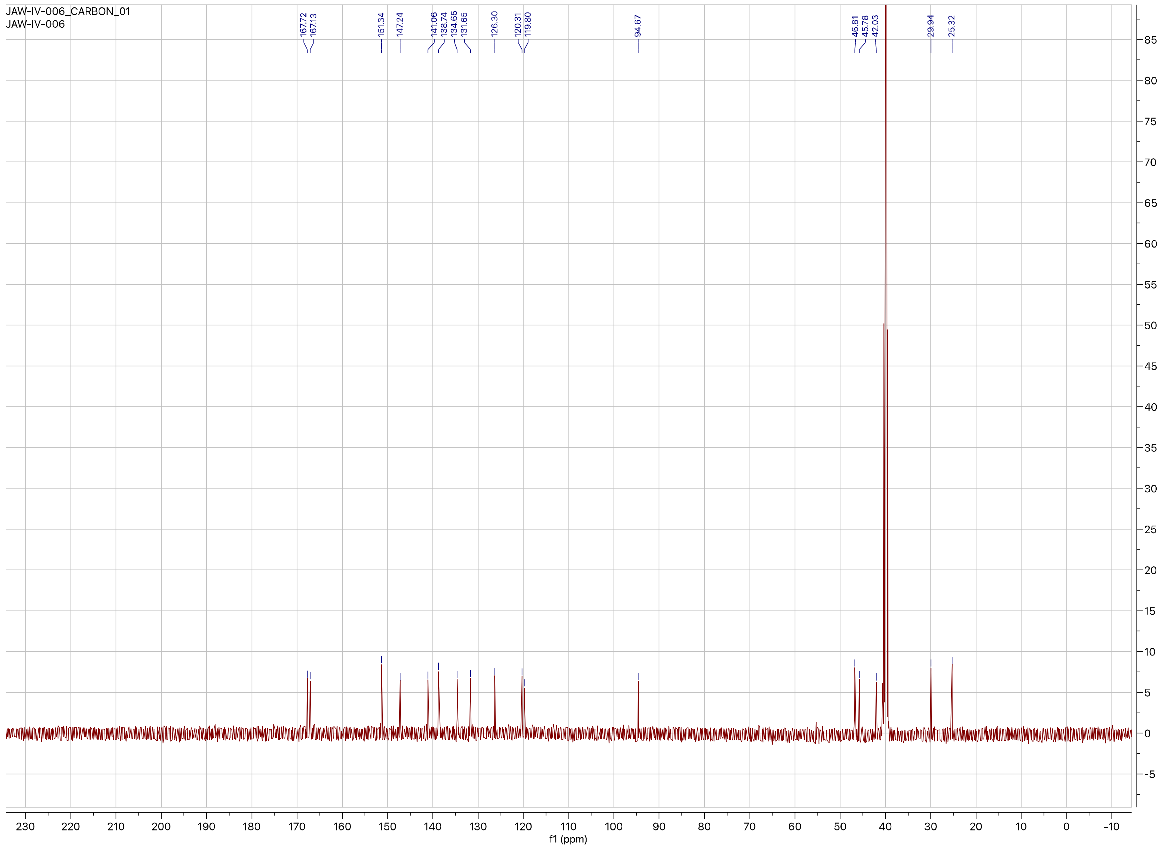

Figure S10. ^{13}C NMR of Compound **6b**.

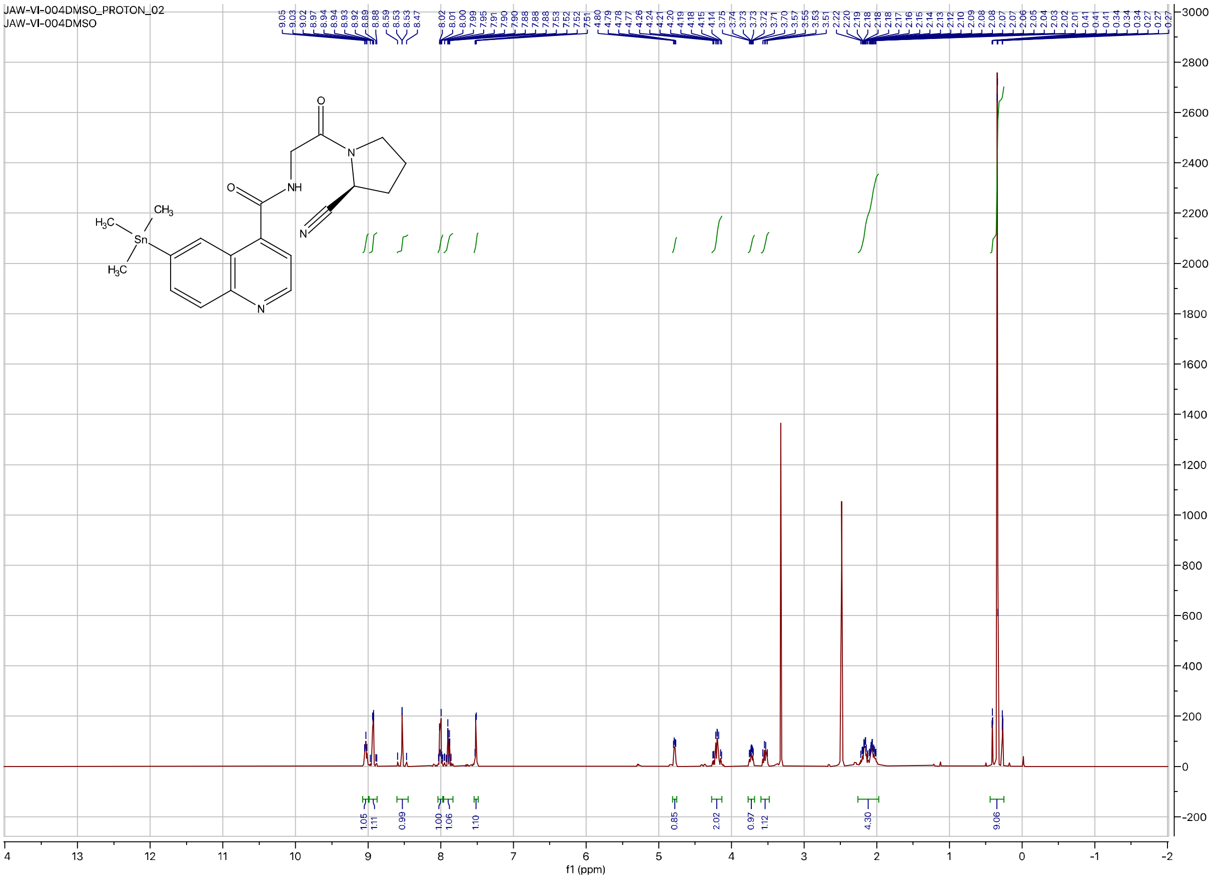

Figure S11. ^{1}H NMR of Me_3SnFAPI **2**.

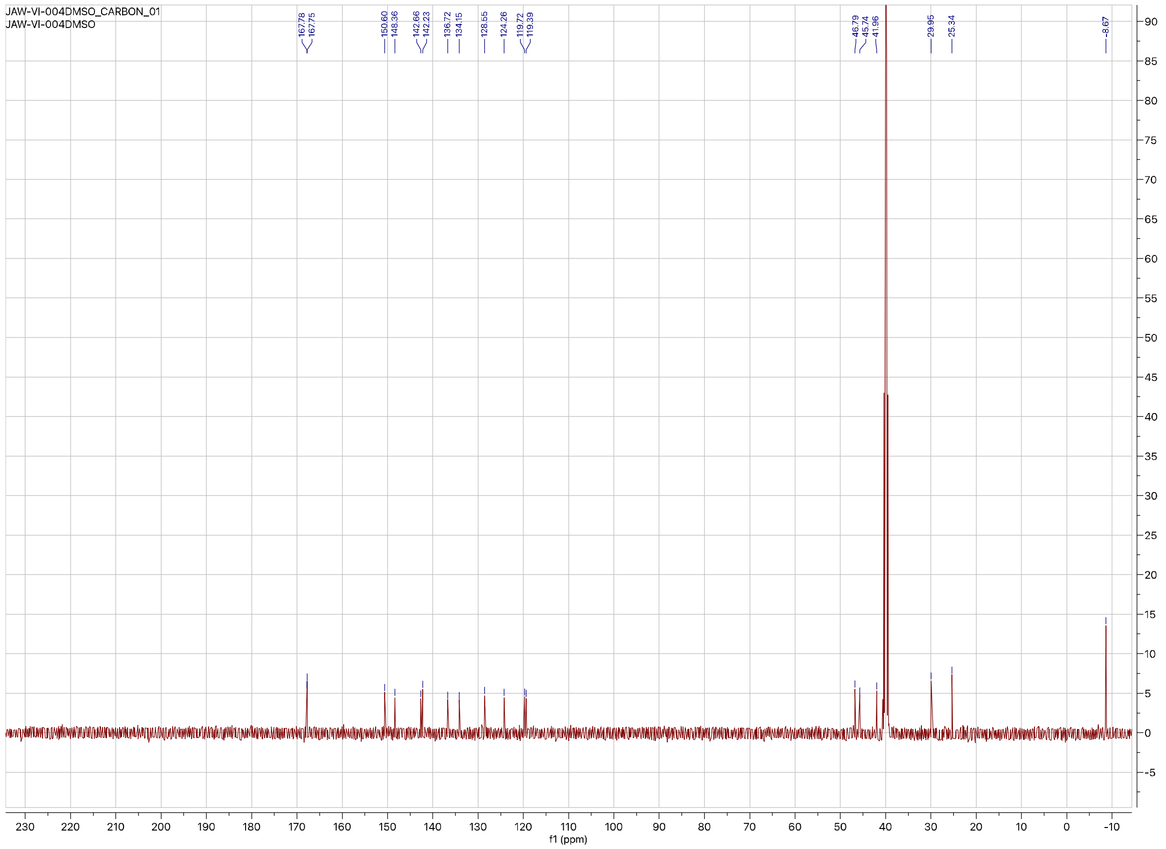

Figure S12. ^{13}C NMR of $Me_3SnFAPI$ **2**.

Solvent: MeOH

Ionization Method: ESI+

Analyzer: TOF

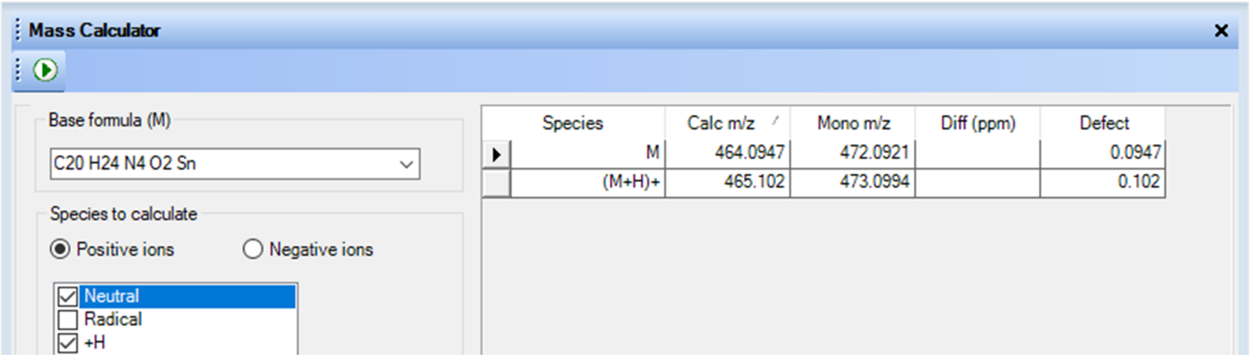

Peak of Interest

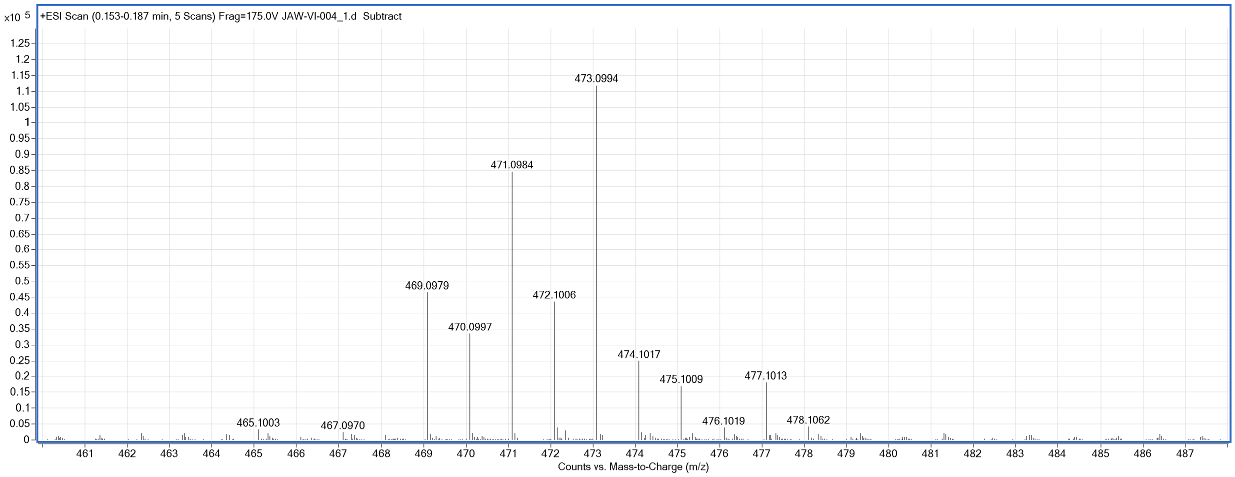

Figure S13. HRMS of $Me_3SnFAPI$ **2**.

2) Radio- and UV-HPLC chromatograms of:

2i) Semi-preparative separation of [^{18}F]FluoFAPI **1**

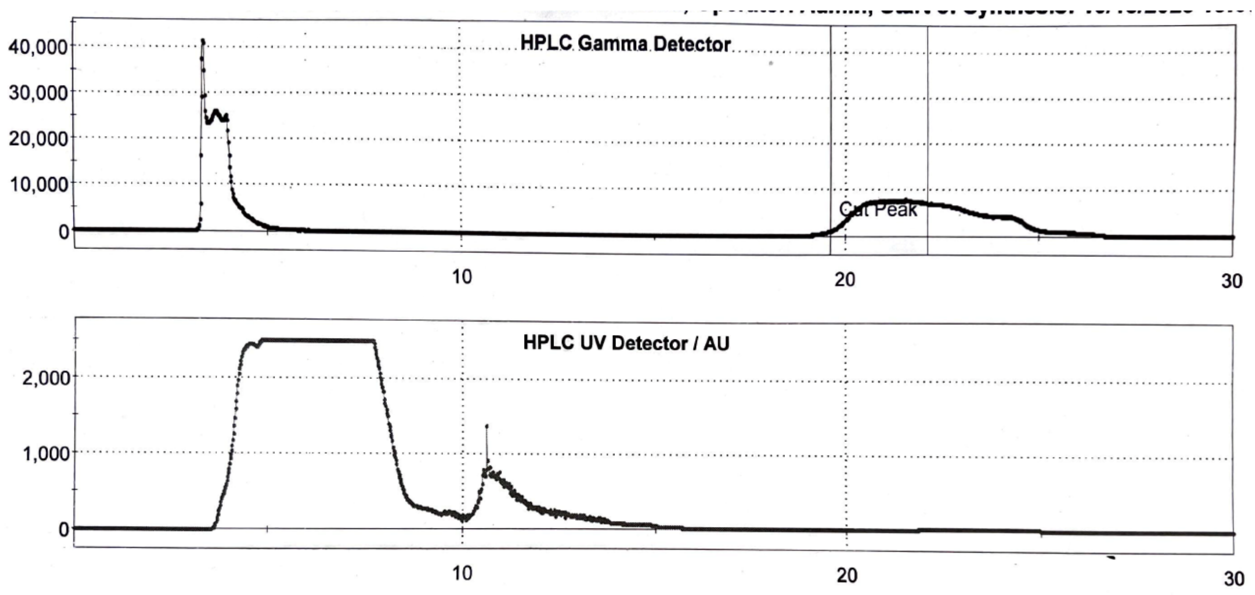

Figure S14. UV- and radio-chromatogram of the semi-preparative HPLC separation of [^{18}F]FluoFAPI **1**.

Column: Gemini 5µ NXC18 110 Å, 250X10; Buffer: 55% MeCN, 10 mM NH_4HCO_3, pH 10.0
Flow rate: 4 ml/min @ rt; UV: 254 nm; Retention time: ~20 min

2ii) Final formulated dose [^{18}F]FluoFAPI **1**

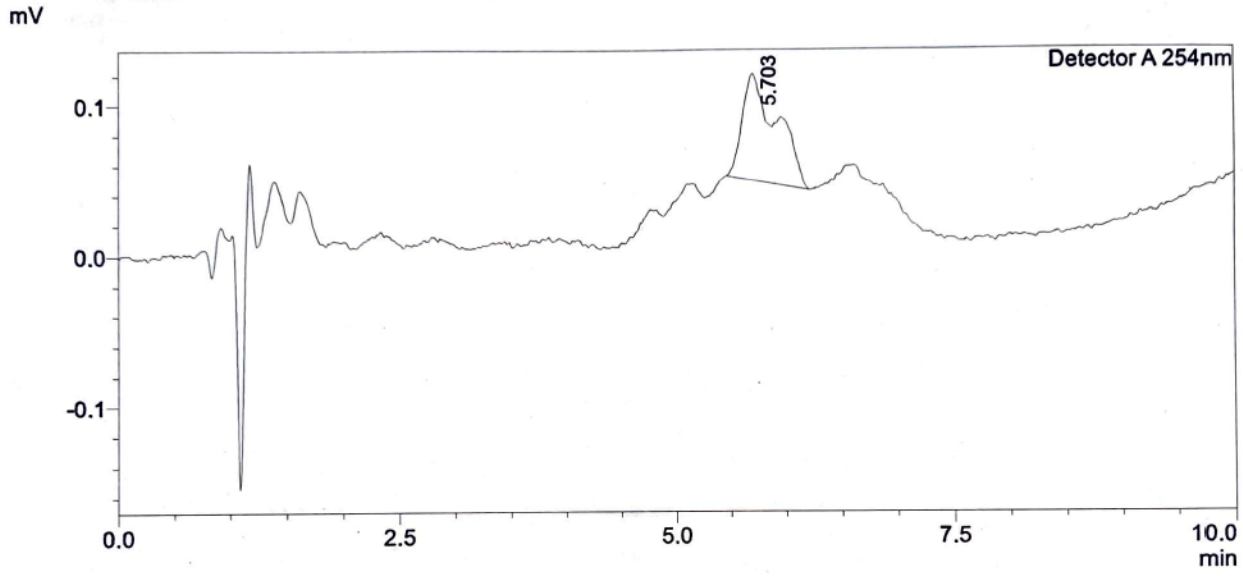

Detector A 254nm

Peak#	Ret. Time	Area	Height	Conc.	Unit	Mark	Name
1	5.703	1545	71	100.000		M	
Total		1545	71				

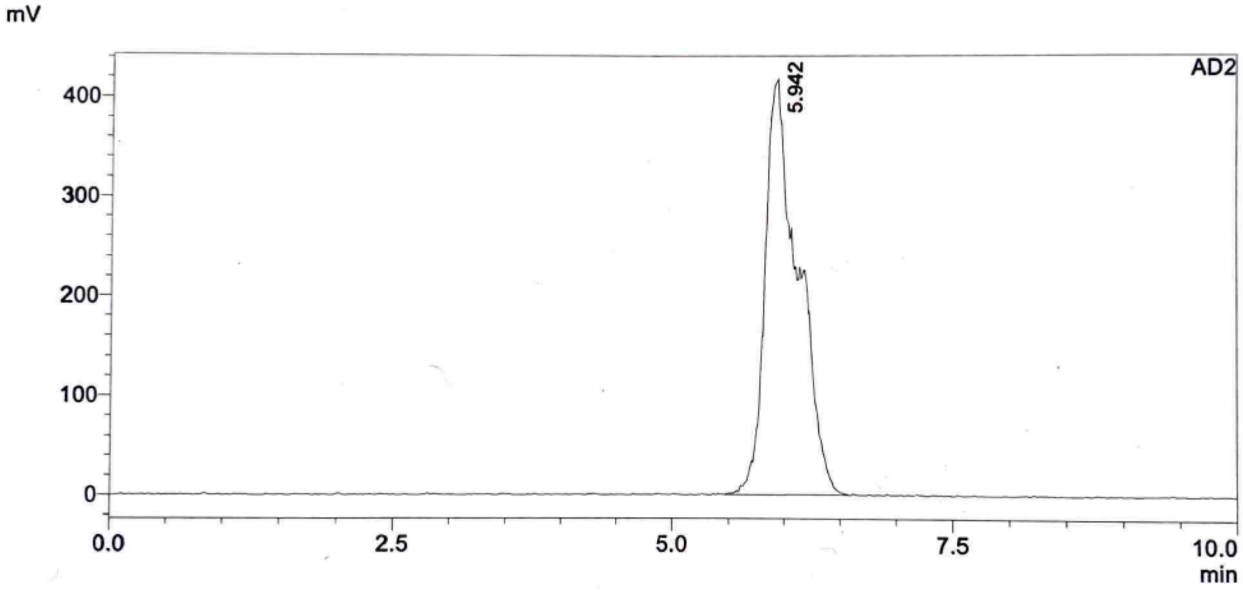

AD2

Peak#	Ret. Time	Area	Height	Conc.	Unit	Mark	Name
1	5.942	8720139	419005	100.000			

Figure S15. Analytical HPLC UV- and radio-chromatogram of [^{18}F]FluoFAPI **1**: Final formulated dose.

Column: Luna 5µ C18(2), 150X4.6; Buffer: 20% MeCN, 10 mM NH_4OAc, pH 5.0 Flow rate: 2 ml/min @ 40 °C; UV: 254 nm; Retention time: ~5.94 min

2iii) Final formulated dose co-inject [^{18}F]FluoFAPI **1**

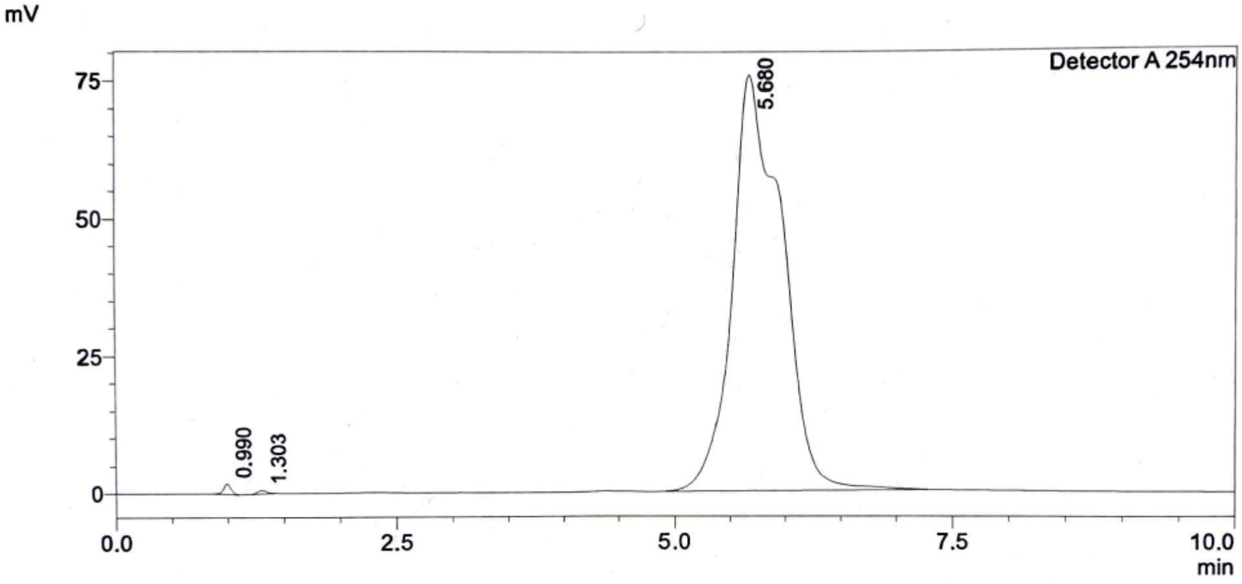

Detector A 254nm

Peak#	Ret. Time	Area	Height	Conc.	Unit	Mark	Name
1	0.990	9090	1979	0.000			
2	1.303	3993	678	0.000			
3	5.680	2412126	75842	0.000			
Total		2425209	78499				

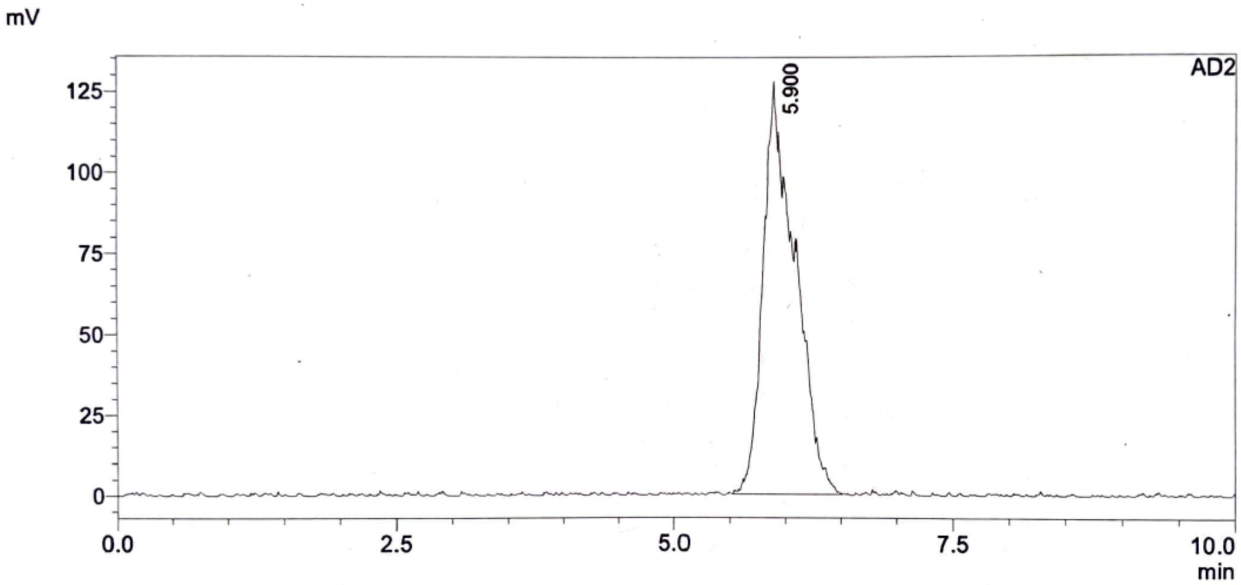

AD2

Peak#	Ret. Time	Area	Height	Conc.	Unit	Mark	Name
1	5.900	2583008	128241	100.000			
Total		2583008	128241				

Figure S16. Analytical HPLC UV- and radio-chromatogram of [^{18}F]FluoFAPI **1**: Co-inject final formulated dose.

Column: Luna 5µ C18(2), 150X4.6; Buffer: 20% MeCN, 10 mM NH_4OAc, pH 5.0 Flow rate: 2 ml/min @ 40 °C; UV: 254 nm; Retention time: ~5.90 min

3) Biodistribution data of [^{18}F]FluoFAPI **1** in Sprague-Dawley rats

Gender Avg Biodistribution

**0.166667**	hrs		
Organ	%ID/g	±	S.E.
Brain	0.0347%	±	0.0067%
Eyes	0.1814%	±	0.0311%
Heart	0.4097%	±	0.0351%
Lungs	0.3438%	±	0.0334%
Liver	0.4987%	±	0.0620%
Pancreas	0.5010%	±	0.0427%
Spleen	0.3239%	±	0.0453%
Adrenals	0.3257%	±	0.0350%
Kidney	0.8577%	±	0.1466%
Adipose	0.0744%	±	0.0101%
Stomach	0.4059%	±	0.0566%
CO Stomach	0.0859%	±	0.0321%
Small Int	0.6419%	±	0.0777%
CO Small Int	0.3234%	±	0.0724%
Caecum	0.3656%	±	0.0713%
CO Caecum	0.1158%	±	0.0239%
Lg Int	0.4403%	±	0.0722%
CO Lg Int	0.1194%	±	0.0408%
Ovaries	0.3103%	±	0.0908%
Uterus	0.3507%	±	0.1027%
Testes	0.0614%	±	0.0011%
Muscles	0.2101%	±	0.0339%
Bone	0.3196%	±	0.0482%
Blood	0.3101%	±	0.0207%
Total	0.3898%	±	0.0594%
Carcass	0.5089%	±	0.0278%

0.333333	hrs		
Organ	%ID/g	±	S.E.
Brain	0.0400%	±	0.0086%
Eyes	0.1791%	±	0.0352%
Heart	0.3099%	±	0.0240%
Lungs	0.2657%	±	0.0187%
Liver	0.3710%	±	0.0308%
Pancreas	0.3607%	±	0.0169%
Spleen	0.2329%	±	0.0199%
Adrenals	0.2649%	±	0.0038%
Kidney	1.1177%	±	0.1473%
Adipose	0.1607%	±	0.0323%
Stomach	0.3653%	±	0.0245%
CO Stomach	0.0596%	±	0.0162%
Small Int	0.5997%	±	0.0634%
CO Small Int	0.3892%	±	0.0121%
Caecum	0.3575%	±	0.0325%
CO Caecum	0.1692%	±	0.0253%
Lg Int	0.3879%	±	0.0384%
CO Lg Int	0.2024%	±	0.0430%
Ovaries	0.3050%	±	0.0043%
Uterus	0.3715%	±	0.0738%
Testes	0.0964%	±	0.0008%
Muscles	0.3269%	±	0.0439%
Bone	0.3713%	±	0.0270%
Blood	0.2647%	±	0.0118%
Total	0.3614%	±	0.0343%
Carcass	0.5322%	±	0.0371%

1	hrs		
Organ	%ID/g	±	S.E.
Brain	0.0139%	±	0.0010%
Eyes	0.0908%	±	0.0073%
Heart	0.1370%	±	0.0027%
Lungs	0.1176%	±	0.0021%
Liver	0.1958%	±	0.0197%
Pancreas	0.1603%	±	0.0007%
Spleen	0.0997%	±	0.0018%
Adrenals	0.1291%	±	0.0032%
Kidney	0.6052%	±	0.1086%
Adipose	0.0933%	±	0.0126%
Stomach	0.2168%	±	0.0353%

CO Stomach	0.2976%	±	0.0343%
Small Int	0.4855%	±	0.0581%
CO Small Int	1.5261%	±	0.1747%
Caecum	0.2338%	±	0.0243%
CO Caecum	0.3415%	±	0.0639%
Lg Int	0.2250%	±	0.0155%
CO Lg Int	0.3062%	±	0.0532%
Ovaries	0.1411%	±	0.0096%
Uterus	0.1758%	±	0.0006%
Testes	0.1006%	±	0.0001%
Muscles	0.1709%	±	0.0045%
Bone	0.1914%	±	0.0106%
Blood	0.1210%	±	0.0058%
Total	0.3460%	±	0.0139%
Carcass	0.3066%	±	0.0186%

**2**	hrs		
Organ	%ID/g	±	S.E.
Brain	0.0077%	±	0.0007%
Eyes	0.0546%	±	0.0102%
Heart	0.0836%	±	0.0075%
Lungs	0.0713%	±	0.0054%
Liver	0.1088%	±	0.0079%
Pancreas	0.0904%	±	0.0081%
Spleen	0.0576%	±	0.0067%
Adrenals	0.0772%	±	0.0065%
Kidney	0.4346%	±	0.1225%
Adipose	0.0692%	±	0.0126%
Stomach	0.1450%	±	0.0236%
CO Stomach	0.2630%	±	0.1404%
Small Int	0.4632%	±	0.0516%
CO Small Int	2.1286%	±	0.6614%
Caecum	0.1925%	±	0.0115%
CO Caecum	0.3539%	±	0.0449%
Lg Int	0.1733%	±	0.0174%
CO Lg Int	0.3408%	±	0.0195%
Ovaries	0.0997%	±	0.0125%
Uterus	0.1131%	±	0.0104%
Testes	0.0690%	±	0.0084%
Muscles	0.1039%	±	0.0088%
Bone	0.1055%	±	0.0119%
Blood	0.0753%	±	0.0050%
Total	0.3529%	±	0.0349%

Carcass	0.2467%	±	0.0186%

Figure S17. Biodistribution data of [^{18}F]FluoFAPI **1** in Sprague-Dawley rats

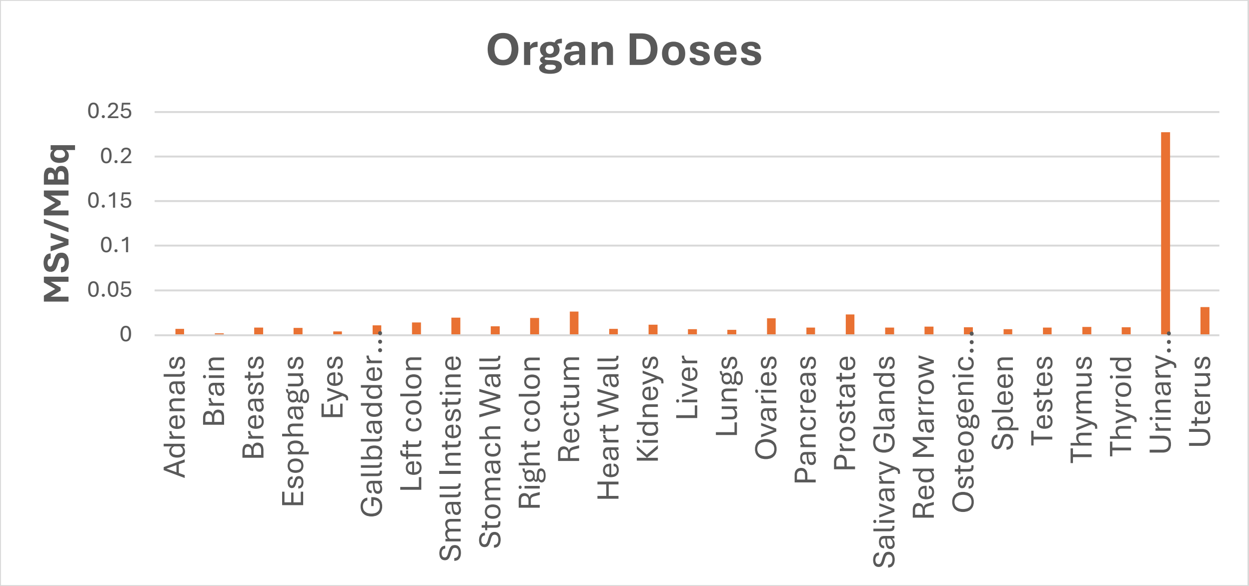

Figure S18. Bar graph of estimated organ doses for [^{18}F]FluoFAPI **1.**

Single Acute Dose Toxicology Data

Figure S19. Sprague Dawley Rat Hematology Changes

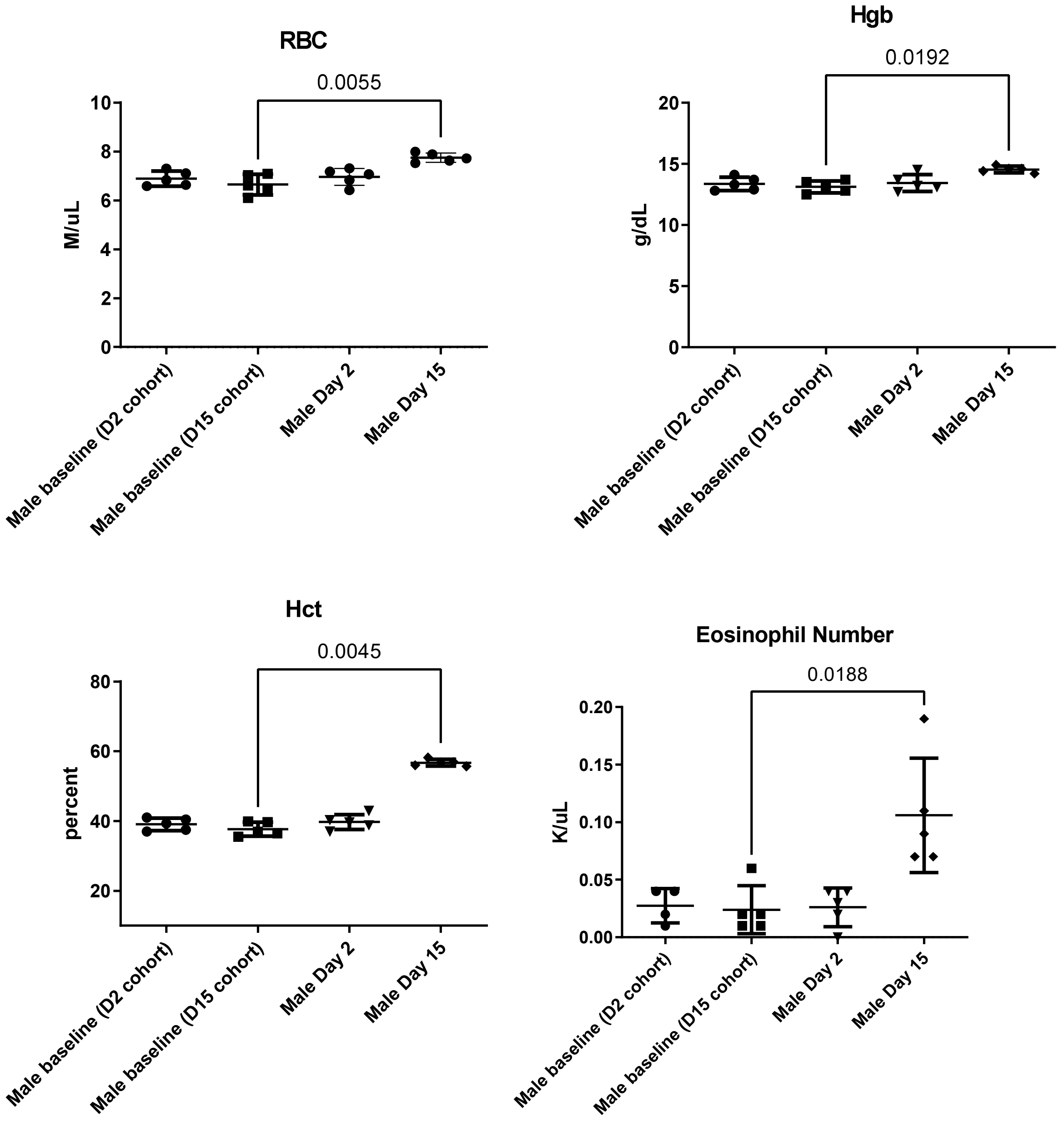

Group	Animal No.	RBC M/uL	Hemoglobin g/dL	Hematocrit %	MCV fL	MCH pg	MCHC g/dL	RDW fL	RDW %
1 Male Day 2 cohort	101	7.31	14.5	42.9	58.7	19.8	33.8	30.7	17.9
	102	6.42	12.7	37.0	57.6	19.8	34.3	30.7	17.1
	103	7.07	13.1	39.6	56.0	18.5	33.1	30.9	19.8
	104	6.83	13.2	38.8	56.8	19.3	34.0	29.5	18.1
	105	7.18	13.7	40.3	56.1	19.1	34.0	30.3	19.3
2 Female Day 2 cohort	201	7.16	13.0	37.4	52.2	18.2	34.8	24.7	17.0
	202	6.89	13.0	37.9	55.0	18.9	34.3	25.8	18.5
	203	7.35	14.1	40.0	54.4	19.2	35.3	25.4	16.5
	204	7.22	13.3	38.7	53.6	18.4	34.4	25.4	17.1
	205	7.53	13.2	37.6	49.9	17.5	35.1	24.9	19.1
3 Male Day 15 cohort	301	7.99	14.6	55.7	53.6	18.3	34.1	27.3	18.7
	302	7.53	14.6	56.9	56.6	19.4	34.3	29.1	17.4
	303	7.89	14.4	56.0	54.0	18.3	33.8	27.8	18.3
	304	7.72	14.9	58.2	56.9	19.3	33.9	27.7	16.4
	305	7.63	14.2	56.8	55.0	18.6	33.6	26.9	16.6
4 Female Day 15 cohort	401	7.42	12.9	36.8	49.6	17.4	35.1	26.2	18.6
	402	7.88	14.4	41.1	52.2	18.3	35.0	25.9	18.0
	403	7.59	14.2	41.4	54.5	18.7	34.3	28.4	18.2
	404	7.61	14.2	40.9	53.7	18.7	34.7	29.1	19.3
	405	7.45	13.5	39.3	52.8	18.1	34.4	26.6	17.9

Group	Animal No.	Reticulocytes K/uL	Reticulocytes %	Platelets K/uL	PDW fL	MPV fL	WBC K/uL	Neutrophils K/uL	Lymphocytes K/uL
1 Male Day 2 cohort	101	570.2	7.80	912	8.0	7.2	10.94	2.92	7.49
	102	582.9	9.08	545	9.3	7.8	9.88	2.09	7.46
	103	581.9	8.23	998	7.5	6.7	8.19	2.54	5.38
	104	532.7	7.80	1061	7.5	6.8	11.84	2.29	9.05
	105	637.6	8.88	1214	8.0	7.1	8.66	1.98	6.38
2 Female Day 2 cohort	201	490.5	6.85	1128	8.8	7.3	7.94	1.74	5.89
	202	478.9	6.95	1049	7.6	6.8	6.59	2.45	3.79
	203	353.5	4.81	1063	8.4	7.1	8.19	1.63	6.27
	204	419.5	5.81	942	7.8	6.8	6.80	1.65	4.78
	205	320.0	4.26	1061	7.5	6.5	8.86	1.54	6.99
3 Male Day 15 cohort	301	239.7	3.00	706	9.7	8.1	6.69	0.91	5.29
	302	279.4	3.71	838	9.1	7.5	6.08	0.81	4.90
	303	288.0	3.65	843	8.1	7.1	5.70	0.87	4.46
	304	271.7	3.52	705	7.8	7.1	11.26	1.60	9.17
	305	288.4	3.78	931	7.4	6.5	6.86	0.93	5.59
4 Female Day 15 cohort	401	238.2	3.21	976	7.2	6.3	4.10	0.86	3.02
	402	302.6	3.84	860	7.8	6.8	8.90	1.45	6.97
	403	266.4	3.51	860	9.3	7.7	4.27	0.56	3.59
	404	265.6	3.49	883	8.3	7.2	4.63	0.86	3.56
	405	248.1	3.33	825	8.6	7.4	4.53	0.53	3.87

Group	Animal No.	Monocytes K/uL	Eosinophils K/uL	Basophils K/uL	Neutrophils %	Lymphocytes %	Monocytes %	Eosinophils %	Basophils %
1 Male Day 2 cohort	101	0.41	0.04	0.08	26.7	68.5	3.7	0.4	0.7
	102	0.27	0.02	0.04	21.2	75.5	2.7	0.2	0.4
	103	0.20	0.03	0.04	31.0	65.7	2.4	0.4	0.5
	104	0.43	0.04	0.03	19.4	76.4	3.6	0.3	0.3
	105	0.27	0.00	0.03	22.9	73.7	3.1	0.0	0.3
2 Female Day 2 cohort	201	0.25	0.05	0.01	22.0	74.2	3.1	0.6	0.1
	202	0.23	0.01	0.02	37.2	57.5	3.5	1.5	0.3
	203	0.20	0.05	0.04	19.9	76.6	2.4	0.6	0.5
	204	0.26	0.05	0.06	24.3	70.3	3.8	0.7	0.9
	205	0.18	0.10	0.05	17.4	78.9	2.0	1.1	0.6
3 Male Day 15 cohort	301	0.38	0.09	0.02	13.6	79.1	5.7	1.3	0.3
	302	0.28	0.07	0.02	13.3	80.6	4.6	1.2	0.3
	303	0.28	0.07	0.02	15.3	78.2	4.9	1.2	0.4
	304	0.27	0.19	0.03	14.2	81.4	2.4	1.7	0.3
	305	0.21	0.11	0.02	13.5	81.5	3.1	1.6	0.3
4 Female Day 15 cohort	401	0.14	0.07	0.01	21.0	73.7	3.4	1.7	0.2
	402	0.35	0.07	0.06	16.3	78.3	3.9	0.8	0.7
	403	0.09	0.02	0.01	13.1	84.1	2.1	0.5	0.2
	404	0.10	0.09	0.02	18.6	76.9	2.2	1.9	0.4
	405	0.05	0.05	0.03	11.7	85.4	1.1	1.1	0.7

Organ Weight (g)						
Group	Animal No.	Brain	Heart	Liver	Spleen	Kidneys (L&R combined)
Males, Day 2	101	**1.917**	**1.104**	**9.864**	**0.860**	**1.751**
	102	**1.893**	**1.070**	**10.642**	**0.874**	**1.924**
	103	**1.962**	**1.158**	**9.298**	**0.715**	**1.925**
	104	**1.930**	**1.091**	**9.175**	**0.736**	**1.856**
	105	**1.866**	**1.042**	**8.860**	**0.496**	**1.914**
Females, Day 2	201	**1.881**	**0.869**	**6.719**	**0.551**	**1.266**
	202	**1.853**	**0.911**	**6.833**	**0.752**	**1.475**
	203	**1.784**	**0.841**	**6.977**	**0.586**	**1.341**
	204	**1.920**	**1.062**	**7.413**	**0.611**	**1.462**
	205	**1.810**	**0.836**	**6.127**	**0.598**	**1.349**
Males, Day 15	301	**1.936**	**1.002**	**9.742**	**0.558**	**1.886**
	302	**1.916**	**0.998**	**11.367**	**0.601**	**1.841**
	303	**2.004**	**1.136**	**11.373**	**0.622**	**2.356**
	304	**1.833**	**1.113**	**11.034**	**0.753**	**2.083**
	305	**1.850**	**1.204**	**10.599**	**0.677**	**2.178**
Females, Day 15	401	**1.951**	**0.921**	**7.882**	**0.536**	**1.428**
	402	**1.640**	**0.894**	**8.595**	**0.572**	**1.461**
	403	**2.011**	**0.863**	**7.790**	**0.610**	**1.588**
	404	**1.902**	**0.947**	**7.852**	**0.675**	**1.569**
	405	**2.050**	**0.838**	**6.481**	**0.660**	**1.384**
